# *Ganoderma lucidum* mediates microglial polarization and ameliorates experimental autoimmune encephalomyelitis by reducing oxidative stress and inhibiting NF-κB/STAT3 pathway

**DOI:** 10.1186/s13020-026-01327-x

**Published:** 2026-04-10

**Authors:** Lu Zhang, Jie Chen, Sauchu Yuen, Meiling Wu, Wenting Li, Shenyu Yan, Jiangang Shen

**Affiliations:** 1 School of Chinese Medicine, State Key Laboratory of Pharmaceutical Biotechnology, LKS Faculty of Medicine, The University of Hong Kong, 3 Sassoon Road, Pokfulam, Hong Kong SAR China; 2 Department of Orthopedics, Shanghai Institute of Traumatology and Orthopedics, Ruijin Hospital, Shanghai Jiao Tong University School of Medicine, Shanghai, 200025 China

**Keywords:** *Ganoderma lucidum*, Microglia polarization, Experimental autoimmune encephalomyelitis, Multiple sclerosis, Nuclear factor-kappa B, Signal transducer and activator of transcription 3

## Abstract

Multiple Sclerosis (MS) is a neuroinflammatory disease affecting the central nervous system (CNS), for which effective therapeutic strategies remain limited. *Ganoderma Lucidum* (GL) is a nature healthy food supplement whose effects on MS remain unknown. Using an experimental autoimmune encephalomyelitis (EAE) mouse model, we tested the hypothesis that GL could mitigate MS by inhibiting microglial activation and promoting a shift toward an anti-inflammatory M2-like phenotype. We demonstrate that early preventive administration of GL significantly reduced disease severity, inflammatory infiltration, demyelination, and microglial activation and polarization by promoting M2-like polarization in the spinal cord of EAE mice. Furthermore, using in vitro cellular experiments, GL revealed antioxidant and anti-inflammatory effects by scavenging superoxide (O_2_·^−^), nitric oxide (NO·) and peroxynitrite (ONOO^−^) and down-regulated the expression of IL-1β, TNF-α, IL-6, COX2, and iNOS in the LPS-stimulated microglial cells. GL inhibited the expression of nuclear factor-κB/signal transducer and activator of transcription 3 (NF-κB/STAT3). Taken together, *Ganoderma lucidum* is a promoting antioxidant and anti-inflammatory healthy supplement to inhibit microglial activation and attenuate the severity and progress of MS pathogenesis by reducing oxidative stress and modulating NF-κB/STAT3 signaling pathways*.*

**Graphical abstract:**

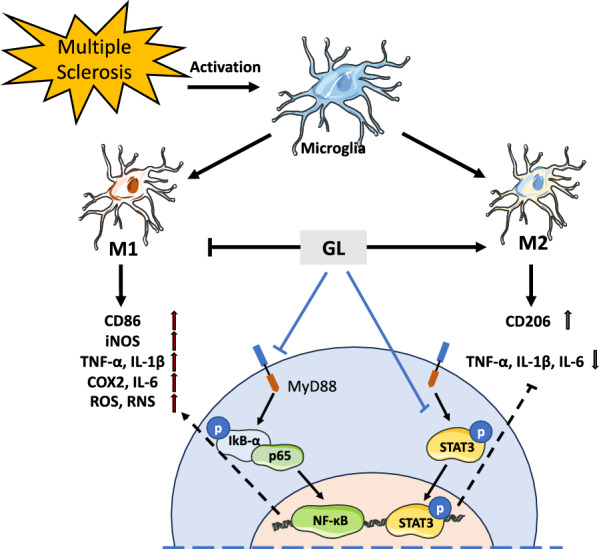

**Supplementary Information:**

The online version contains supplementary material available at 10.1186/s13020-026-01327-x.

## Introduction

Multiple Sclerosis (MS) is an autoimmune neurodegenerative disease characterized by neuroinflammation, demyelination, gliosis, and neurodegeneration in the central nervous system (CNS), leading to motor disability and cognitive dysfunction, etc. [10–12, 33]. Although current therapeutic strategies alleviate symptoms and modulate disease relapse, long-term use often results in side effects and drug resistance [9]. Moreover, no drug can reverse the disease’s progress [38]. Thus, developing novel prevention and therapeutic approaches for MS treatment is desirable.

Experimental autoimmune encephalomyelitis (EAE) is a well-established animal model with the key features of MS, providing a valuable tool for drug discovery and mechanistic studies [37]. In the EAE model, inflammatory cells infiltrate the CNS, triggering excessive production of reactive oxygen species (ROS) and reactive nitrogen species (RNS), including NO·, O_2_·^−^, ONOO^−^, H_2_O_2_, and OH [30]. ROS/RNS leads to oxidative and nitrative stress, causing lipid peroxidation, protein oxidation, oxidative damage, and neuronal death [7, 25]. Macrophages and microglia are major sources of ROS/RNS during MS progression [35], making microglia-generated oxidative stress a potential pharmacological target for alleviating MS.

Microglia, the resident macrophages of the CNS, are essential for regulating myelin growth and associated cognitive function as well as preserving myelin integrity by preventing its degeneration [28]. However, microglia are rapidly activated in neurodegenerative diseases [23, 34]. The lymphocyte-microglia-astrocyte axis plays crucial roles in mediating white matter inflammation and degeneration during chronic active multiple sclerosis [1]. Activated microglia and astrocytes produce pro-inflammatory cytokines, further recruiting inflammatory cells and exacerbating inflammation and demyelination [22]. Conversely, reduced microglial activation or microglial paralysis suppresses EAE progression [17]. At the early stages of EAE/MS pathogenesis, residential microglia and macrophages infiltrated from peripheral tissues are expressed as the M1 phenotype that release pro-inflammatory cytokines, leading to tissue damage in the CNS. While at the late stage, the M2 phenotypes for resolving inflammation and tissue repair become predominant in the CNS. Transition and polarization from the pro-inflammatory M1 phenotype to the regulatory or anti-inflammatory M2 phenotype can restore homeostasis and improve neurological functional outcomes [8]. Thus, targeting microglial activation and polarization represents a promising strategy to attenuate MS pathogenesis.

*Ganoderma lucidum* (GL), commonly known as “Lingzhi”, is a medicinal mushroom that has been used in traditional Chinese medicine (TCM) for centuries. Until now, over 100 triterpenoids and polysaccharides have been identified from its fruit body, mycelium, sporangium powder, and fermentation broth, demonstrating anti-inflammatory, anti-tumor, hypoglycemic, and neuroprotective effects [16, 20, 32]. *Ganoderma lucidum* polysaccharides (GLP) reveal the bioactivities of anti-neuroinflammation and neuroprotection through modulation of microglial inflammatory and behavioural responses [4]. GLP alleviates cognitive dysfunction by inhibiting neuroinflammation via the NLRP3/NF-κB signaling pathway [21]. With the potential neuroprotective and anti-inflammatory properties, GL holds promising values as a healthy supplement for neurodegenerative disorders and neuroinflammatory diseases. However, few studies are available to support the use of *Ganoderma lucidum* for MS treatment.

In this study, we tested the hypothesis that *Ganoderma lucidum* (GL) powder could reduce inflammatory infiltration, promote remyelination and alleviate neural deficit symptoms in the EAE mouse model. The underlying mechanisms could be associated with its antioxidant and anti-inflammation properties by inhibiting microglial activation.

## Materials and methods

### Reagents

*Ganoderma Lucidum* (GL) powder, named Lingzhi Vitality Capsules, was provided by Beijing Tong Ren Tang Chinese Medicine Co. LTD; it consists of both *Ganoderma Lucidum* fruitbody and *Ganoderma Lucidum* Spore Powder. Lipopolysaccharides (LPS) from Escherichia coli O111:B4 and 3-(4,5-dimethylthiazol-2-yl)−2,5-diphenyltetrazolium bromide (MTT) were purchased from Sigma-Aldrich Company (St. Louis, MO, USA). JSH-23 (CAS No.: 749886-87-1), the inhibitor of NF-κB, were purchased from MCE to monitor the inhibitory effect of GL. Fluorescent latex beads (1 µM, L2778, Sigma) were used for phagocytosis determination. Cell surface-staining antibodies were obtained from eBioscience (San Diego, CA, USA), including Brilliant Violet 421™ anti-mouse CD206 (C068C2), and FITC anti-mouse CD86 (GL-1). Primary antibodies and secondary antibodies were purchased from the companies including: CD 206 (ab64693, Abcam), iNOS (ab3523, Αbcam), CD86 (ab119857, Abcam), Phospho-NF-κB p65 (#3033, CST), NF-κB p65 (#8242, CST), β-actin (#3700, Cell Signaling), COX2(sc1747, Santa Cruz), IL-6 (ab290735, Αbcam), IL-1β(sc-52012, Santa Cruz), TNF-α (11948 T, CST), Anti-mouse antibody (7076 s, Cell Signaling), Anti-rabbit antibody (7074 s, Cell Signaling), Anti-goat antibody (#559,286, BD biosciences). For HPLC or LC–MS/MS detection, all solvents used were HPLC-grade. For RNS-released level determination, the Nitric Oxide Assay Kit reagent was purchased from Abcam (ab272517, Abcam). 4,5-diaminofluorescein diacetate (DAF-2 DA; Sigma, USA), and hydroethidine (HEt, Sigma, USA) were purchased to determine the production of NO· and O_2_·^−^ respectively, HK Yellow-AM were used to determine the intracellular ONOO^−^level obtained from Prof. Yang DAN’s group (Chemical Biology, HKU, HK). To determine the toxicity of GL in mouse liver and kidney, plasma commercial kits was utilized (Z003-1-1 & Z002-1-1, Nanjing Jiancheng Co., Ltd.).

### Preparation of GL powder extract

GL powder was weighed at 10 g and mixed with 150 ml of 70% ethanol (EtOH) to facilitate extraction. The mixture was heated for 30 min and then cooled to room temperature. The liquid extract was separated and collected, while the solid residue was collected to repeat extraction. The extraction procedure was repeated for a total of three cycles. The collected liquid portion was evaporated using a rotary evaporator to get the concentrated extract; then, a freeze-drying apparatus was utilized to get a dried extract. The extraction yield was calculated using the formula: Extraction Yield (%) = (Weight of dried extract/Weight of spore powder) × 100%.

### EAE induction and drug treatment

C57BL/6N mice were purchased from the Centre for Comparative Medicine Research (CCMR), HKU. All experiments were reviewed and approved by the Committee on the Use of Live Animals in Teaching and Research (CULATR, 4721-19).

Active experimental autoimmune encephalomyelitis (EAE) mouse models were induced as our previously reported [24]; eight- to ten-week-old mice were adopted and immunized subcutaneously with 200 µg myelin oligodendrocyte glycoprotein_35–55_ (MOG_35–55)_ incomplete Freund’s adjuvant supplemented to 5 mg/ml of Mycobacterium tuberculosis. Pertussis Toxin (200 ng, Sigma-Aldrich) was injected twice on day 0 and day 2 post-immunization (dpi) intravenously. Daily assessments of body weight and clinical scores were processed. The clinical scores of EAE mice were evaluated according to the following standards: 0, no clinical signs; 0.5 partially limp tail; 1. decreased tail tone; 1.5 weak in hindlimb or lost coordinated movement; 2, ineffective in hindlimb with lost cooperative movement; 2.5 one hindlimb paralyzed; 3, both hindlimbs paralyzed; 4, forelimb paralysis, hindlimbs paralyzed; 5, moribund.

For drug administration, the GL powder was suspended or dissolved in Milli-Q water and orally administered to the immunized EAE mice daily. To evaluate and optimize the potential of GL as a health supplement for the prevention or treatment of MS, three experimental protocols were adopted, including early pretreatment protocol, prevention protocol and treatment protocol. For the early pretreatment protocol, the mice were treated with GL for 7 days before EAE induction; for the prevention protocol, the mice were treated from 2 days until 30 days post-immunization (dpi); for the treatment protocol, the GL was administered from disease onset (around 10 dpi) to 30 dpi. The vehicle was treated with an equal volume of Milli-Q water as a control.

The GL raw material, provided by Beijing Tong Ren Tang Co., Ltd., with an official recommended human dosage of 6 capsules per day (350 mg per capsule) with a standard body weight of 60 kg. Mouse dosages were calculated using the Meeh-Rubner interspecies conversion formula, as adopted in the method described by Yang et al. [42]: $Dosage_{mouse} = \, Dosage_{human} \times \left( {K_{mouse} /K_{human} } \right)$, where the $K_{mouse} = 1$ and $K_{human} = 0.11$, with the following calculations: The low, middle and high dosages (0.11, 0.32, 0.96 mg/g) were orally administrated to the mice, equivalent to 2, 6 and 18 capsules of human daily dosages respectively.

### Histopathology

For histopathology determination, the mice at 18 dpi and 30 dpi were sacrificed and perfused with PBS. The lumbar spinal cord (L4-L6) was collected and fixed with 4% paraformaldehyde (PFA). The isolated tissue was dehydrated, permeabilized, and then embedded with paraffin. After embedding, the tissues were cut into 5 µm sections and stained with Hematoxylin & Eosin (H&E) or Luxol Fast Blue (LFB) to assess the inflammatory infiltration or the demyelination in the spinal cord. The scoring system was defined as previously described [25]. The levels of inflammatory infiltration were scored as follows: 0, no inflammatory infiltration; 1, a few inflammatory cells around blood vessels; 2, slight inflammatory cells infiltrate into parenchyma; 3, few inflammatory cells infiltrate into parenchyma with extension into the adjacent tissue; 4, many inflammatory cells infiltrate into parenchyma with a large amount of adjacent tissue influenced. For the demyelination level stained by LFB, the evaluation standards were given as follows: 0, no demyelination observed; 1, rare demyelination foci in the pia mater; 2, obvious subpial and perivascular demyelination; 3, confluent areas if demyelinating plaque in subpial or perivascular areas; 4, extensive subpial and perivascular demyelination with large number of inflammatory cells in the CNS parenchyma.

### Toxicology

C57BL/6N mice (8–10 week) were utilized for toxicology determination. Mice were randomly divided into health control group, GL treatment group, EAE group and GL treated EAE group for determination (n = 3–4). All animals were orally treated saline or GL (0.96 mg/g once per day, which is equal to 18 capsules for human) once per day for 10 consecutive days or 30 consecutive days. Animal body weight is collected and recorded daily. All the animals were sacrificed at day 11(disease onset day) for blood collection and tissue collection. The liver and kidney were separated for histopathology determination for the drug toxicity test. The liver and kidney function were determined by using serum of blood with the centrifuge at 3000 g * 15 min at 4 ˚C by a refrigerated centrifuge, and the plasma Alanine Aminotransferase(ALT), plasma Aspartate Aminotransferase (AST), plasma Creatinine (Cr), plasma Blood Urea Nitrogen (BUN) and plasma Uric Acid (UA) levels using commercial kits (Z003-1-1 & Z002-1-1, Nanjing Jiancheng Co., Ltd.) with data recorded by microplate reader (Model 680, Bio-Rad) according to manufacturer’s guidance. The liver and kidney damage were determined by H&E staining method using optical microscopy (Olympus, NY, USA).

### Immunofluorescence and immunohistochemistry

For immunofluorescence (IF) and immunohistochemistry (IHC) processing, dissociated tissues were embedded into O.C.T or paraffin, cut into 30 µm or 5 µm sections, and stored at – 20 ˚C. Tissue slices were processed and retrieved for 15 min in 10 mM sodium citrate buffer (pH = 6.0) at 95 ˚C, followed by PBS washing three times. The 3% H_2_O_2_ was added to slides for the IHC sample preparation. For staining, the slides or cells were blocked with 5% goat serum for 1 h at RT for IF/IHC staining, followed by the primary antibodies staining at 4 ˚C overnight. After being washed with PBST, the secondary antibodies conjugated with fluorochrome or HRP were used to stain for another 2 h at room temperature. For IF staining, the DAPI was used to stain the nucleus for 15 min and mounted with an antifade medium (Dako); samples were captured using the confocal microscopy LSM 880/900 (Carl Zeiss). For IHC staining, the DAB kit was followed by hematoxylin, mounted with Canada Balsam, and examined by light microscopy (Carl Zeiss).

### Cell culture and lipopolysaccharides (LPS) treatment

Mouse BV2 microglial and PC12 cells were obtained from American Type Culture Collection (ATCC, Manassas, VA). The BV2 and PC12 cells were cultured in high-glucose Dulbecco’s Modified Eagle Medium (DMEM) supplemented with 10% heat-inactivated fetal bovine serum (FBS, Gibco), 1% penicillin/streptomycin (PS, Gibco), and 1% 2 mM L-glutamine (Gibco). These culture conditions provided the necessary nutrients and support for the growth and maintenance of BV2 and PC12 cells.

For subculture, both BV2 and PC12 cells were collected using trypsin digestion and then passaged at a split ratio of 1:3 according to ATCC’s guidelines. This subculture method ensured the proper maintenance and propagation of the cells, allowing for their continued growth and use in subsequent experiments. Those cells were collected and seeded into a 24-well Transwell plate at a density of 2 × 10^4^ cells per well (PC12 cells), 5 × 10^4^ cells per well for a 24-well plate or 2 × 10^4^ cells per well for a 24-well Transwell plate or 1 × 10^5^ cells per well for a 12-well plate (BV2 cells), and cultured for 24 h until they reached approximately 80% confluency. The BV2 cells were pretreated with GL extraction (70% ethanol, 200 μg/ml) with or without JSH-23 inhibition (30 µM) for 1 h. Then LPS (2 µg/mL) was added to the BV2 cells and incubated for 24 h. After achieving the designed experiments, the cells were collected for further analysis.

### Flow cytometry

The flow cytometry method was adopted to identify the subtypes of microglial cells. The cells were digested by using Trypsin and separated into single cells. For identifying microglial polarization, after being washed with PBS, the cells were stained by incubating with BV421-conjugated anti-CD206 and FITC-conjugated anti-CD86 (1:100) for 1 h in the dark at room temperature. All stained cells were then examined using the ACEA Novocyte Quanteon instrument (Agilent, CA, USA) at a flow rate of 0.8 mL/min and calculated using FlowJo software (Treestar, Ashland, USA).

### Western blot analysis

Protein was collected by incubating with RIPA plus 100:1 protease inhibitor cocktail (Sigma-Aldrich) (v/v) for 30 min. The protein was resolved in 10% SDS–polyacrylamide gels and transferred to 0.45 µm PVDF membranes. After blocking by 5% BSA in TBST, the membrane was reacted with different primary antibodies including iNOS(1:1000), p-p65(1:1000), p65(1:1000), β-actin(1:1000), TNF-ɑ 1:1000), IL-1β (1:1000), iNOS (1:1000), COX2 (1:1000), MyD88 (1:1000), p-IκBα (1:1000), STAT3 (1:1000), and p-STAT3 (1:1000) for 24 h, followed by incubation with HRP-conjugated secondary antibodies (1:2000–5000) for 4 h. Then the ECL reagents were used to determine the interest proteins, and the result was captured by the Gel-Doc system (Bio-Rad, CA, USA).

### Real-time PCR

Real-time PCR was performed to quantify the expression levels of target genes. A SupreReal Premix Plus SYBR Green kit (Thermo Fisher Scientific, USA) was used for PCR reactions along with sequence-specific primers listed in Table 1. The SupreReal Premix contained all necessary components in each reaction, including SYBR Green I dye, DNA polymerase, dNTPs, and reaction buffers. Template cDNA was added, and the reaction plate was placed in the instrument. The qPCR was conducted on a Lightcycler 480 II instrument. The program began with a 15-min denaturation step at 95 °C to activate the polymerase enzyme. This was followed by 40 cycles of two-step amplification, with 10 s of denaturation at 95 °C and 20 s of annealing/extension at 55 °C. Fluorescence was detected at the extension step of each cycle. At the end of the cycling, melting curve analysis was performed to validate the specific amplification of single PCR products. Relative expression levels for target genes were calculated using the 2-ΔΔCt method, with normalization to the endogenous GAPDH and 18 s for reference genes.

**Table 1:** Primers used in real-time PCR

Target gene	Sequence (5′−3′)	
	Forward	Reverse
18S	GCAATTATTCCCCATGAACG	GGCCTCACTAAACCATCCAA
IL-6	CCTTCTTGGGACTGATGCTGGTG	AGGTCTGTTGGGAGTGGTATCCTC

### Phagocytosis

BV2 cells were cultured in 24-well plates to determine the phagocytic ability. GL (200 mg/mL) was administered for 1 h, followed by LPS stimulation (2 µg/mL. After being treated for 24 h, the culture medium was gathered and switched to incubate with DMEM for 30 min. The fluorescent latex beads (1 µM, L2778, Sigma) were pre-activated in 50% fetal bovine serum (FBS) in PBS for the phagocytosis assay. Then, the beads were added into respective wells at a concentration of 100 beads per BV2 cell, followed by incubating for 3 h and calculating using a cell counter (Countess 3, Invitrogen). Then, the cells were washed 3 times with PBS and fixed with 4% PFA at room temperature for 10 min. Images were captured using a confocal microscope LSM 880 or LSM 900 (Carl Zeiss), with 405 and 561 nm wavelengths. The phagocytotic levels were also assessed using flow cytometry, specifically by utilizing the FITC channel.

### MTT assay

PC12 cells were seeded in the 24-well Transwell cell culture plates at a density of 5 × 10^4^ cells/well at the lower compartment cocultured with BV2 cells at the upper compartment at a density of 2 × 10^4^ cells/well for cell viability analysis. Cells were cultured for 24 h for growth and then challenged with LPS for 24 h. For the treatment group, drugs were administered 1 h before LPS. After treatment, MTT (5 mg/ml) was added to each well to generate formazan crystals in intact cells. After 4 h of incubation, dimethyl sulfoxide (DMSO, 800µL) was added to dissolve the formazan. The absorbance was measured at 570 nm by a multi-plate reader (Model 680, Bio-Rad) and calculated.

$${\text{Cell viability}}\% \, = \, \left( {{\mathrm{OD}}_{{{\mathrm{experiment}}}} - {\mathrm{OD}}_{{{\mathrm{blank}}}} / {\mathrm{OD}}_{{{\mathrm{control}}}} - {\mathrm{OD}}_{{{\mathrm{blank}}}} } \right){\text{ x1}}00\%$$

### LC-MS/MS analysis

Chemical profiles of GL powder were identified by using an LC–MS/MS spectrometer integrated with Waters Auto-Purification LC/MS Systems, which include an Autosampler and collector (2767), a Makeup pump (515), a System fluidic organizer, Photodiode array (PDA) detector (2998), pump (2545) and a Qda mass detector. Component separation was achieved on an ACQUITY UPLC^®^ BEH C18 column (1.7 µm). The specific chromatographic conditions were as follows: a sample injection volume of 5 µL at a concentration of 10 µg/mL, a flow rate of 0.6 mL/min, a column temperature of 35.0 °C, and a sample temperature of 20.0 °C. The mobile phases consisted of A (0.1% formic acid in water) and B (0.1% formic acid in acetonitrile). The optimized gradient profile was set up as follows: from 0 to 2 min at 28% B; from 2 to 5 min, increasing from 28 to 35% B; from 5 to 26 min, from 35 to 43% B; from 26 to 30 min, from 43 to 80% B; from 30 to 35 min, from 80 to 95% B; from 35 to 50 min, holding at 95% B.

For the MS/MS analysis, mass detection was conducted in negative electrospray ionization (ESI) mode with the following operational parameters: declustering potential (DP) set at − 80 V with the DP spread set as 0 V, capillary temperature maintained at 300 °C, accumulation time of 0.25 s, and a scan range of 100–1000 m/z. Nitrogen was utilized as the sheath gas at a pressure of 30 units and as auxiliary gas at 5 units. The in-source collision potential energy was adjusted to − 10 V to facilitate the dissociation of dimers or sodium adducts, while the automatic gain control (AGC) settings were appropriately configured.

### Statistical analysis

All data were gathered and measured for at least three replicates. Unpaired Student’s t-test for two groups was designed, and one-way ANOVA followed by Duncan’s multiple range test was used for multiple group comparisons. Data were expressed as the Mean ± S.E.M. All analyses were performed using GraphPad Prism for Mac V8.0 software (GraphPad Software Inc., CA, USA). Probability (P) values < 0.05 were considered statistically significant.

## Results

### Quality control analysis of GL

The chemical constituents of GL were qualitatively analyzed using liquid chromatography-tandem mass spectrometry (LC–MS/MS). In negative ion mode (Supplementary Fig. 1, Fig. S1), 19 compounds were successfully identified by matching diagnostic ions and fragmentation pathways with reference compounds [3, 6]. A summary of the identified compounds, including their fragment ions and retention time, is provided in Supplementary Table 1. A total of 19 identified compounds were classified as ganoderic acids, highlighting the chemical profile of the GL extract.

### GL powder, with early pretreatment and prevention protocols, alleviates EAE progression and severity in active EAE mice

We then evaluated the effects of GL powder on neurological deficit scores in the mouse EAE model. Mice were allocated into the EAE plus vehicle treatment, EAE plus GL treatment, and EAE plus dimethyl fumarate (BG-12) positive treatment groups. To evaluate the GL’s prophylactic and treatment effectiveness, we adopted three protocols, including the early pretreatment protocol treated for 7 days before immunization, the prevention protocols for day 2 to day 30 post-immunization, and the treatment protocols for day 11 (onset day) to day 30 of post-immunization (dpi). We first tested three dosages of GL (0.11, 0.32, and 0.96 mg/g/day) using the prevention protocol. Notably, the 0.32 mg/g/day dosage showed optimal efficacy to ameliorate maximum and cumulative clinical scores (Fig. S2). Therefore, the dosage of 0.32 mg/g/day was selected for subsequent experiments.

**Fig. 1:**
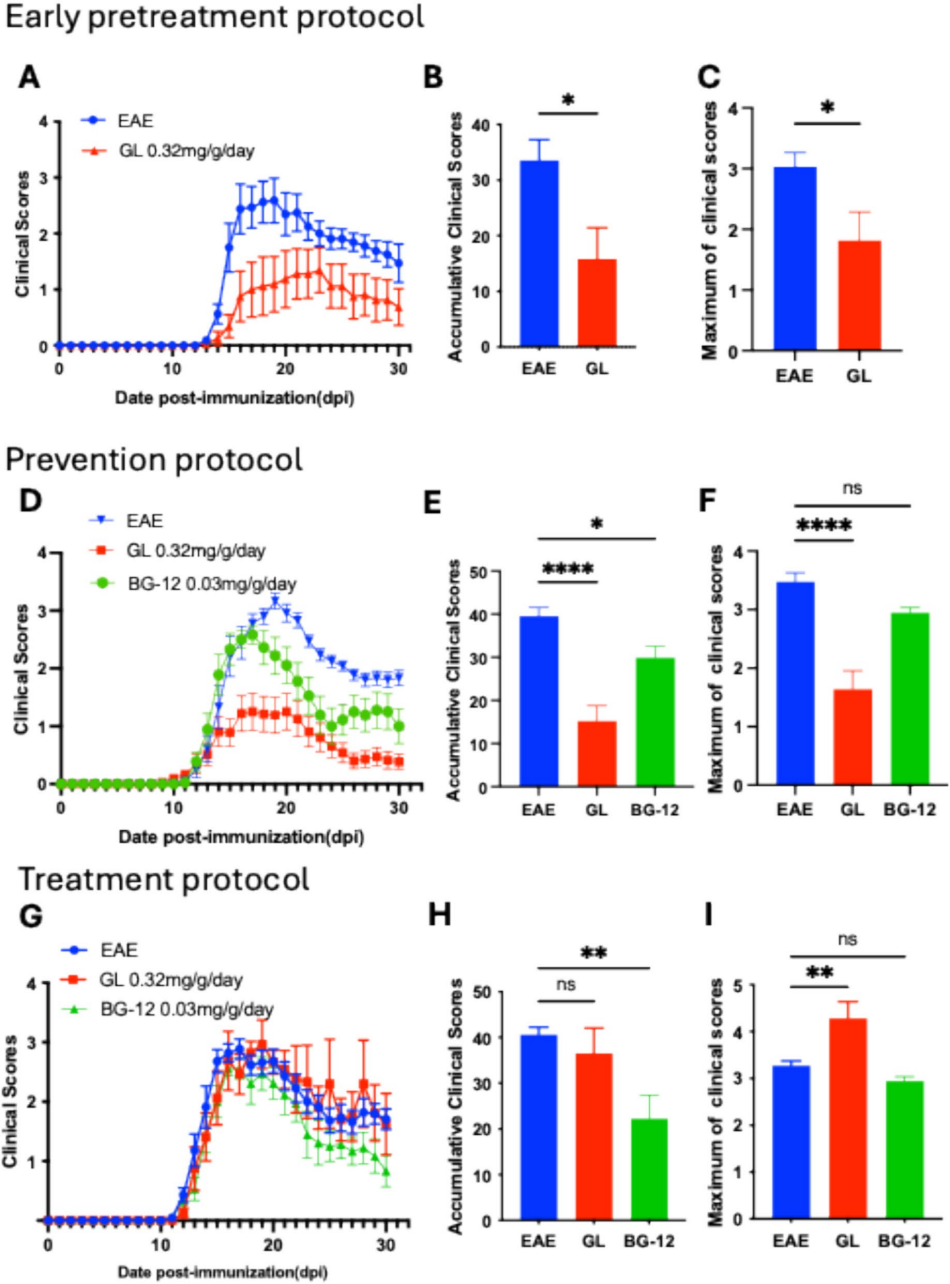
GL mitigates active EAE symptoms with early pretreatment protocol and prevention protocol, but not in treatment protocol. Mice were orally administrated with vehicle (n = 8) or GL powder (0.32 mg/g/day, n = 8–12) daily with three different protocols including early pretreatment, prevention and treatment starting at 7 days prior to EAE induction (**A–C**), day 2 post EAE induction (**D–F**) and disease onset **(G–I**), respectively. Accumulative clinical scores for 30 dpi and the maximum clinical scores were calculated. The data are presented as mean ± S.E.M., with statistical significance indicated by *p < 0.05, **p < 0.01, or ****p < 0.0001 relative to the vehicle-treated EAE group. Statistical analysis was performed using unpaired Student’s t-test (**B, C**) or one-way ANOVA (**E, F, H, I**)

Active EAE mice with the vehicle treatment revealed mild clinical signs like tail and hind limb weakness at the onset stage and progressed to peak symptoms with hind limb paralysis at 16–20 dpi. With both early pretreatment protocol and prevention protocol, GL treatment (0.32 mg/g/day) significantly delayed the peak time of disease severity, alleviated EAE symptoms (Fig. 1A, D), and decreased cumulative and maximum clinical scores (Fig. 1B, C, E, F) in comparison with the vehicle EAE group. Meanwhile, in the positive control group, the pretreatment of BG-12 (0.03 mg/g/day, from day 2 to day 30 post-immunization) administered into the same regimen of the EAE mice significantly attenuated cumulative clinical scores but had no significant difference in maximum clinical scores in comparison with EAE vehicle treatment (Fig. 1D–F). Interestingly, with the treatment protocol, GL treatment had no significant effect on disease progression. Neither maximum nor cumulative clinical scores showed improvement (Fig. 1G, H, I). Those results suggest that GL could be a prophylactic healthy food supplement rather than a therapeutic agent for MS treatment.

### GL powder, with early pretreatment and prevention protocols, attenuates neuroinflammation and demyelination in the CNS of EAE mice

**Fig. 2:**
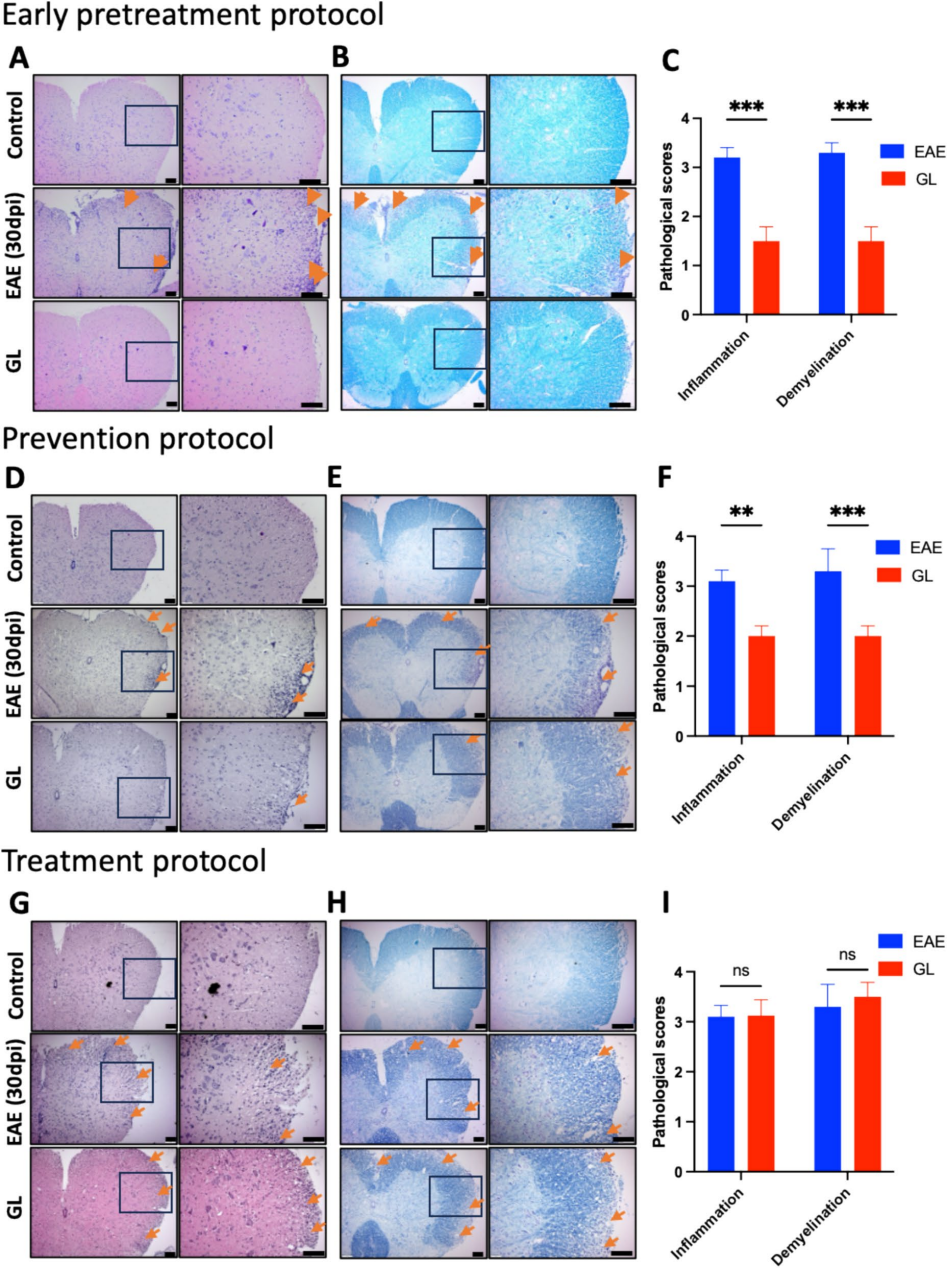
GL treated with early pretreatment protocol and prevention protocol rather than treatment protocol ameliorates inflammation and demyelination in EAE mice. Mice were allocated into the groups of sham control (control), EAE plus vehicle control (EAE), and EAE plus GL (GL, 0.32 mg/g/day). Representative H&E staining (**A, D, G**) and Luxol Fast Blue (LFB) staining (**B, E, H**) showed inflammation and demyelination respectively in lumbar spinal cords (L4-L6, 30 dpi) from EAE mice treated with early pretreatment protocol (**A–C**), prevention protocol (**D–F**) and treatment protocol (**G-I**) (Scale bars, 100 µm). Statistical results (**C, F, I**) revealed that GL treatment with an early pretreatment and prevention protocol, reduces inflammation and demyelination compared to the treatment protocol (**D, F, I**). Values are presented as Mean ± S.E.M. (n = 4), *p < 0.05, **p < 0.01, and ***p < 0.001, relative to the vehicle-treated EAE group. Statistical analysis was performed using One-way ANOVA

To evaluate anti-inflammation and neuroprotective effects, we next performed hematoxylin and eosin (H&E) staining and Luxol fast blue (LFB) staining on spinal cord tissues of active EAE mice that were collected on 30 dpi. The vehicle control EAE mice revealed inflammatory infiltration and demyelination in the lesions of the lumbar spinal cords, as shown by H&E and LFB staining, respectively (Fig. 2). The H&E staining showed dense blue dots at the L4–L6 spinal cord segments in the vehicle-treated EAE group, indicating the extensive inflammatory infiltration. The LFB staining showed large light blue areas, indicating demyelination, in the white matter of the same spinal cord segments in the vehicle-treated EAE group. Demyelination, the loss of the myelin sheath, disrupts normal nerve function and contributes to neurological deficits. While the treatment of GL powder significantly reduced the inflammatory infiltration and demyelination when administered under early pretreatment and prevention protocols (Fig. 2), consistent with the clinical scoring results, GL had no effects on improving inflammatory infiltration or demyelination when administered under the treatment protocol. These results suggest that GL could attenuate neuroinflammation and demyelination when administered with an early pretreatment and prevention protocol rather than a treatment protocol.

### Prophylactic treatment of GL reduces CD3^+^, CD11b^+^, and CD45^+^ cell populations and suppresses inflammatory infiltration into lumbar spinal cords in active EAE mice

Next, we adopted a prevention protocol to explore anti-inflammation and neuroprotective mechanisms. The samples were collected at the 11, 18 and 30 dpi, corresponding to the EAE onset, peak time and chronic phases, respectively (Fig. S3 A). Immunohistochemistry (IHC) was performed to identify infiltrated leukocytes in the L4–L6 spinal cord of active EAE mice by using an anti-CD45 antibody (Fig. S3 B). The IHC staining revealed that GL prophylactic treatment significantly reduced CD45^+^ leukocyte infiltration into the spinal cord compared with the vehicle-treated EAE group, indicating its ability to mitigate inflammatory processes and reduce CNS damage. We performed immunofluorescence staining to further characterize the infiltrated leukocyte populations (Fig. S4). Compared to the vehicle EAE group, GL treatment significantly reduced the intensity and distribution of CD3^+^ T cells, CD11b + macrophages/microglia, and CD45^+^ leukocytes, confirming its anti-inflammatory effects on active EAE mice.

### GL powder exerts has no hepatotoxicity and nephrotoxicity

We evaluated potential hepatotoxicity and nephrotoxicity in healthy mice and active EAE mice following intragastric administration of GL (0.96 mg/g) for 10 or 30 consecutive days. GL treatment did not alter body weights and had no hepatic or renal damage, as confirmed by H&E staining (Fig. S5A, S5B). With high-dose GL treatment for 10 days, those mice remained serum and liver enzyme levels within normal ranges. Renal function markers (CRE, BUN, UA) also showed no significant changes compared to controls. Long-term safety assessment at 30 days post-induction (dpi) revealed no significant differences in serum ALT, AST, BUN, or UA across healthy, untreated EAE, and GL-treated EAE mice. Serum CRE was slightly reduced in untreated EAE mice versus healthy controls (p < 0.05), but no notable difference was found between GL-treated and untreated EAE mice. Histopathological evaluation demonstrated intact liver and kidney architecture without signs of inflammation or injury in GL-treated EAE mice. Together, these results indicate that GL administration at the tested dose does not induce significant hepatotoxicity or nephrotoxicity and supports its long-term safety in this model.

### GL powder attenuates microglial activation and promotes M2-like phenotype formation in spinal cords of active EAE mice

**Fig. 3:**
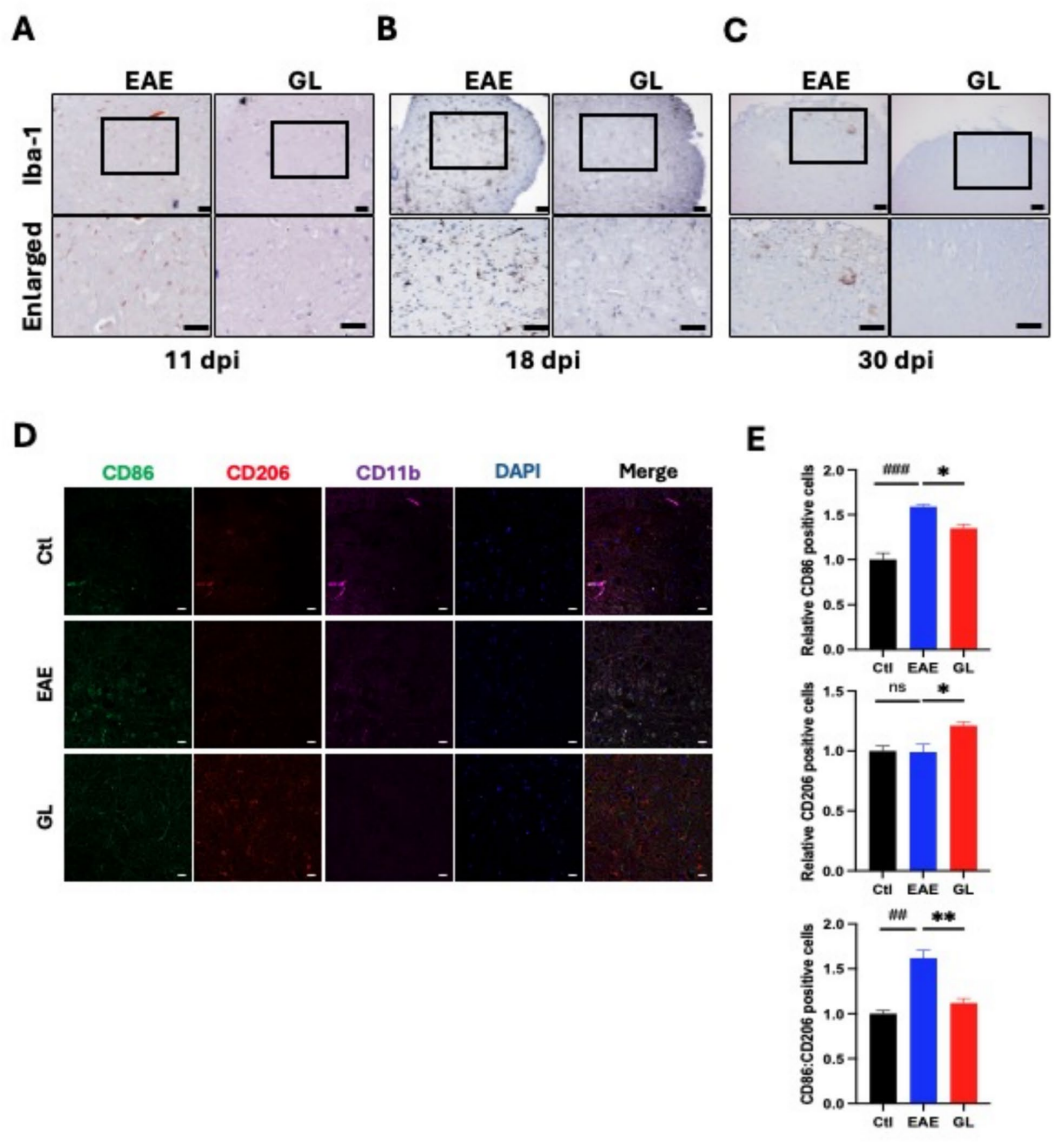
GL attenuates microglial activation and polarization in spinal cords of EAE mice. EAE mice were treated with GL (0.32 mg/g/day, prevention protocol) or vehicle. Tissue samples of spinal cords (L4—L6) were collected and compared at 11 dpi (**A**), 18 dpi (**B**), and 30 dpi (**C**). **A–C**: Representative immunohistochemical (IHC) assessing microglial activation using the Iba-1 marker. EAE, EAE plus vehicle; GL, EAE plus GL. Upper: original IHC staining with highlight area (scale bar = 50 µm); Lower: Enlarged imaging of the highlight area. GL revealed to inhibit microglial activation at all disease phases. **D–E**: Immunofluorescent (IF) staining of microglial polarization in spinal cords (L4–L6) at 30 dpi. CD86 is used for M1-type marker, CD206 for M2-type marker, and CD11b for microglia marker. Ctl: sham control; EAE: EAE plus vehicle; GL: EAE plus GL. Representative IF staining imaging (**D**). Statistical results for relative populations of CD86-positive or CD206-positive cells co-stained with CD11b (**E**). All data are presented as Mean ± S.E.M. (n = 4), ##p < 0.01, ###p < 0.001, versus the control group; *p < 0.05, **p < 0.01, versus the EAE group. Statistical analysis was performed using One-way ANOVA followed by the Newman-Keuls test

**Fig. 4:**
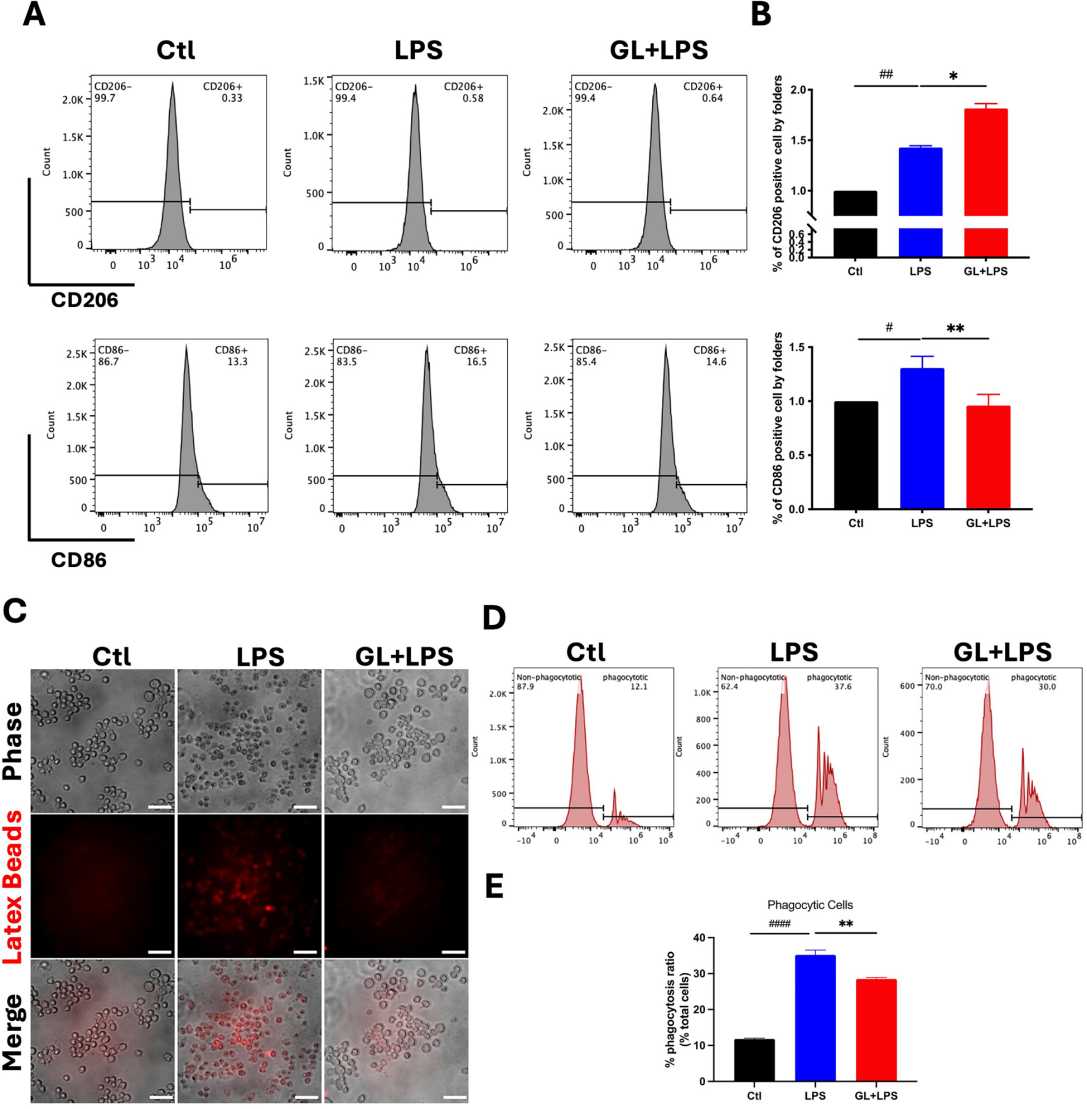
GL extract attenuates microglial polarization and phagocytic capacity in LPS-stimulated BV2 cells. BV2 cells were pre-incubated with GL 70% EtOH extraction (200 µg/ml) for 1 h prior to LPS (2 µg/ml) challenge for 24 h. **A** Representative flow cytometry results show the M1 to M2 polarization shift using FITC-CD86 (M1-like marker) and BV421-CD206 (M2-like anti-inflammatory marker). **B** Statistical analysis on flow cytometry analysis (n = 3). **C** Immunofluorescent staining on Latex beads for phagocytic capacity in LPS-stimulated microglial cells. **D** Flow cytometry detection for Latex beads for phagocytic capacity in LPS-stimulated microglial cells. **E** Statistical analysis on phagocytic capacity in LPS-stimulated microglial cells (n = 4, scale bars, 50 µm). All data are represented as Mean ± S.E.M. #p < 0.05, ##p < 0.01, ####p < 0.0001 versus control group, **p < 0.01, **p < 0.01 versus LPS group. Statistical analysis was performed using one-way ANOVA followed by the Newman-Keuls test

To explore anti-inflammatory mechanisms of GL, we assessed microglial activation in the spinal cords of EAE mice using Iba-1 immunohistochemistry. Tissue was collected at 11, 18, and 30 dpi, representing the onset, peak, and chronic disease stages, respectively. Prophylactic GL treatment significantly reduced microglial activation at all time points (Fig. 3A–C). This suppression was pronounced during the onset (11 dpi) and peak (18 dpi) phases compared to vehicle-treated controls (Fig. 3A, B), indicating an early intervention in the inflammatory cascade. The inhibitory effect persisted into the chronic phase (30 dpi) (Fig. 3C), demonstrating sustained activity. By using in vivo immunofluorescence and in vitro flow cytometry, we determined microglial polarization. In EAE mice, GL treatment significantly decreased expression of the M1 marker CD86 while increasing expression of the M2 marker CD206 (Fig. 3D), suggesting a shift from a pro-inflammatory to an anti-inflammatory phenotype. This was further confirmed in cultured microglia by flow cytometry, where GL exposure reduced the proportion of M1-like cells and increased that of M2-like cells (Fig. 4A, B). Collectively, these data demonstrate that GL modulates microglial activation and promotes a phenotypic shift toward the anti-inflammatory M2 state, which likely contributes to its neuroprotective effects.

### GL powder inhibits lipopolysaccharide (LPS)-induced microglial phagocytosis and reveals antioxidant and anti-inflammatory activity through down-regulating NF-κB/STAT3 pathways in vitro

**Fig. 5:**
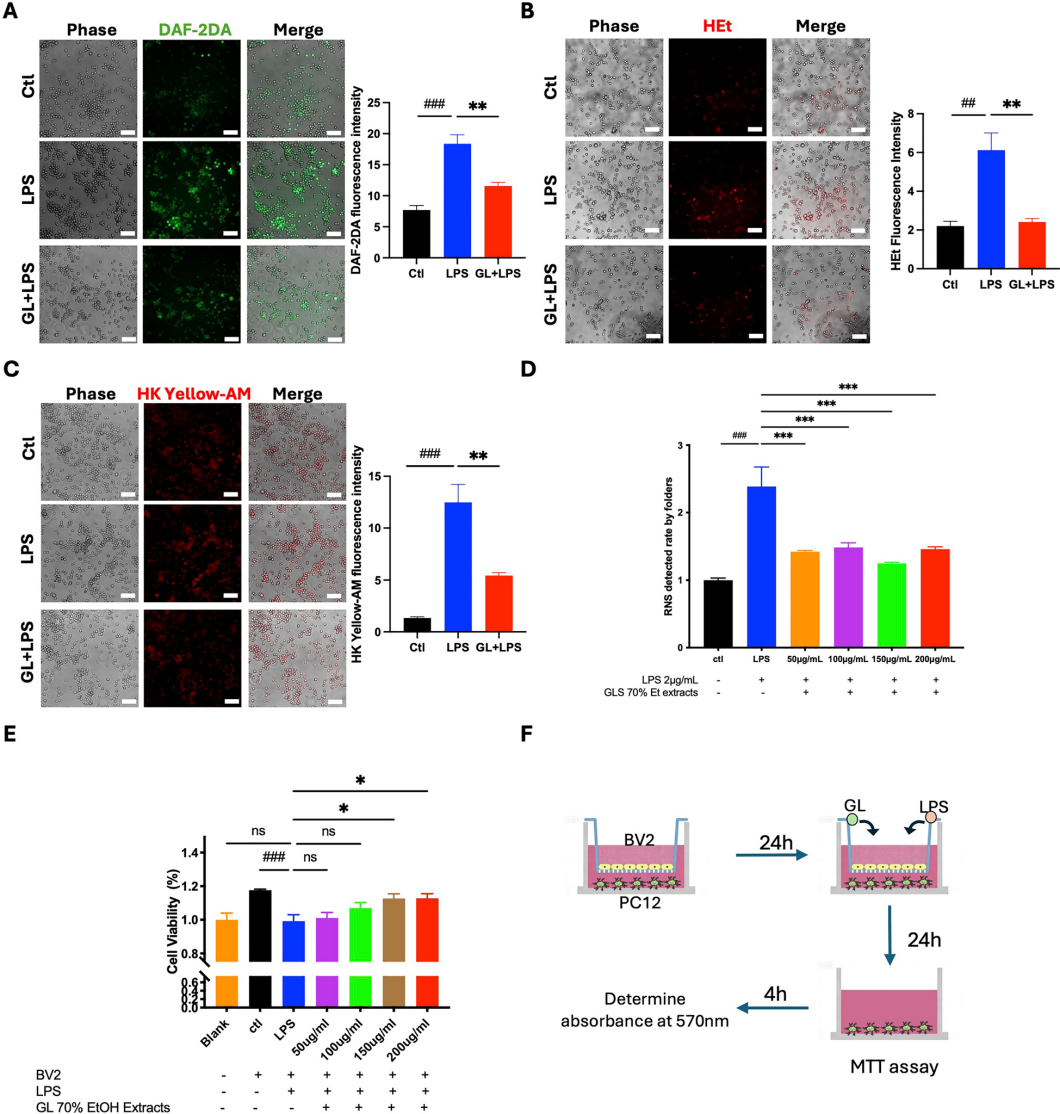
GL extract reduces intracellular NO·, O_2_·^−^, and ONOO^−^ levels in BV2 microglial cells in vitro (n = 3, scale bars, 50 µm). BV2 cells were pretreated for 1 h with GL 70% EtOH extraction (200 µg/ml) followed by 2 μg/mL LPS stimulation for 24 h. **A** Immunofluorescent imaging for NO· production using DAF-2 DA probe. **B** Immunofluorescent imaging for intracellular O_2_·^.−^ production using the HEt probe. **C** Immunofluorescent imaging for intracellular ONOO- production using HK Yellow-AM probe. **D** Total RNS levels in the cell culture medium were detected using an RNS detection kit. GL was demonstrated to decrease the accumulation of NO·, O_2_·^−^, ONOO^−^ and total RNS levels in the BV2 cells. **E** MTT assay for cell viability. **F** Experimental protocol: Microglial cells co-cultured with PC12 for 24 h, then pretreated with GL 70% ethanol extraction 1 h prior to LPS stimulation for another 24 h followed by MTT determination. Data are presented as Mean ± S.E.M. #p < 0.05, ###p < 0.001, versus blank/control group, **p < 0.01, ***p < 0.001, versus LPS group. Statistical analysis was performed by using one-way ANOVA followed by the Newman-Keuls test

**Fig. 6:**
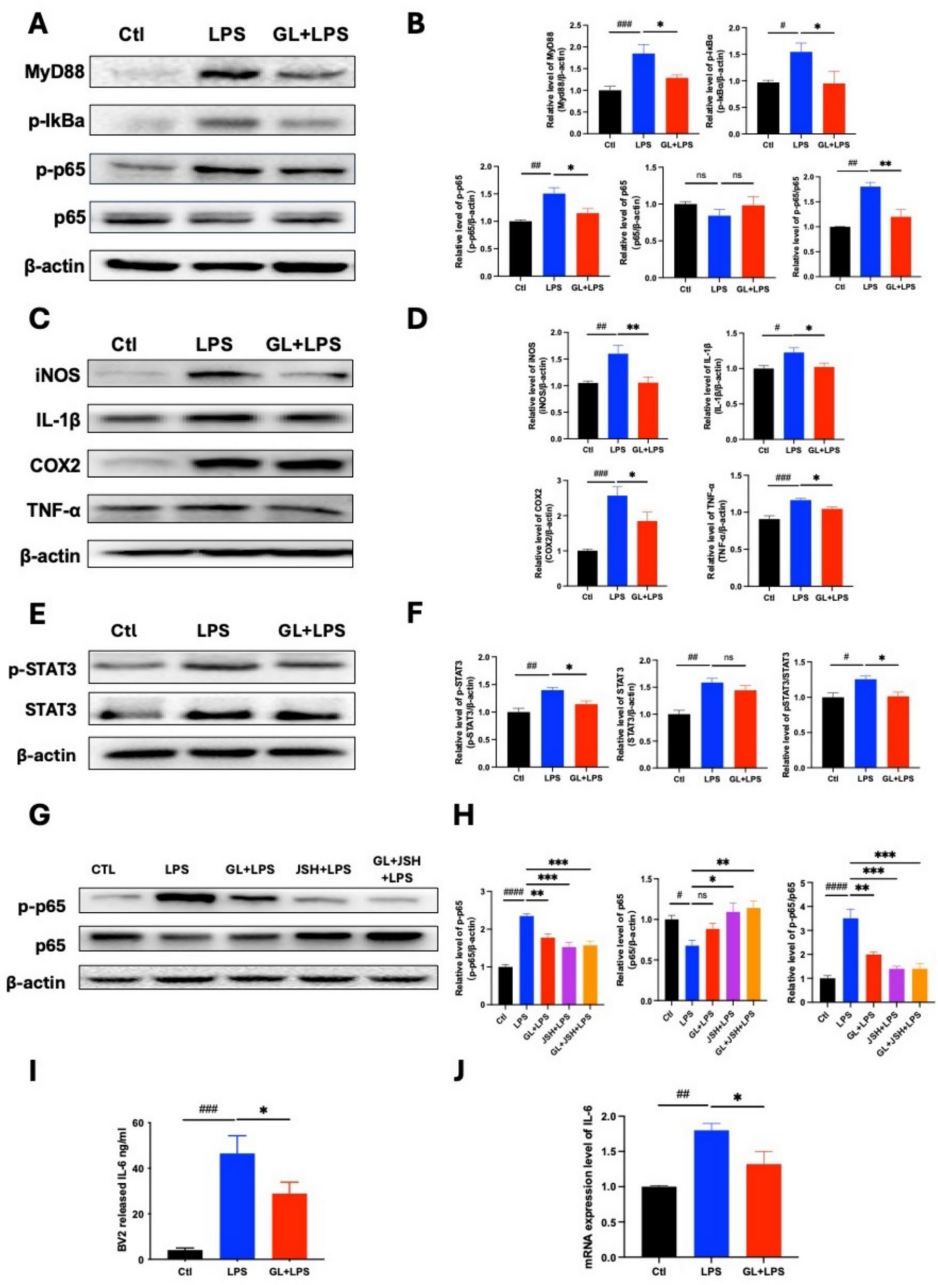
GL extract reduces LPS-stimulated microglial inflammation through NF-κB and STAT3 pathways. BV2 microglial cells were pre-incubated with GL 70% EtOH extraction (200 μg/ml), with or without the NF-κB inhibitor JSH-23 (30 µM) for 1 h prior to LPS stimulation at 2 μg/ml for 24 h. **A** Representative Western blot results on the expression of MyD88, p-IκBα, P-p65, and p65. **B** Statistical analysis on MyD88, p-IκBα, P-p65, and p65. **C** Representative Western blot analysis on IL-1β, TNF-α, COX2, and iNOS expression. **D** Statistical analysis on IL-1β, TNF-α, COX2, and iNOS. **E** Representative Western blot analysis on the expression of pSTAT3 and STAT3. **F.** Statistical analysis on pSTAT3 and STAT3. **G** Representative Western blot analysis on the expression of p-p65 and p65 with NF-κB inhibitor JSH-23 treatment. **H** Statistical analysis on p-p65 and p65 with JSH-23 treatment. **I** ELISA detection of IL-6 level in culture medium. **J** qPCR detection of the expression of IL-6 mRNA in microglial BV cells. Data are shown as Mean ± S.E.M., #p < 0.05, ##p < 0.01, ###p < 0.001, and ####p < 0.0001 versus control group, *p < 0.05, **p < 0.01, ***p < 0.001, ****p < 0.0001 versus LPS group. Statistical analysis was performed using one-way ANOVA followed by Newman-Keuls test (n = 3)

Activated microglia display enhanced phagocytic activity, which can intensify neuroinflammation and neuronal damage in EAE/MS [18]. Regulating this phagocytic response is therefore critical to limit disease progression. We assessed microglial phagocytosis by measuring the uptake of fluorescent latex beads in vitro (Fig. 4C–E). LPS stimulation increased the phagocytic rate in BV2 microglial cells from approximately 20–45%. Treatment with a GL ethanol extract (70% EtOH) significantly reduced the phagocytic rate. Activated microglia can also induce oxidative stress by generating reactive nitrogen species (RNS), contributing to neuronal damage [36]. We examined the effect of GL on the levels of superoxide anion (O₂·⁻), nitric oxide (NO·), and peroxynitrite (ONOO⁻) in LPS-stimulated BV2 cells using fluorescent probes (HEt, DAF-2 DA, and HK-Yellow AM, respectively). GL ethanol extract significantly decreased intracellular levels of O₂·⁻, NO·, and ONOO⁻ (Fig. 5A–C). Consistent with this, a general RNS detection assay confirmed that GL significantly inhibited total RNS production (Fig. 5D). Furthermore, GL ethanol extract significantly suppressed the LPS-induced phosphorylation of p65 and STAT3, and downregulated the expression of pro-inflammatory mediators IL-1β, COX2, TNF-α, IL-6, and iNOS (Fig. 6A–F, I, J). Immunofluorescence analysis further showed that GL inhibited the nuclear translocation of p65 in stimulated BV2 cells (Fig. S6), supporting its inhibitory effect on NF-κB activation. These findings suggest that GL exerts antioxidant and anti-inflammatory effects in LPS-stimulated microglia, in part by inhibiting the NF-κB and STAT3 signaling pathways. To further confirm the role of the NF-κB pathway, we used the specific inhibitor JSH-23. Both GL extract and JSH-23 mitigated the LPS-induced increase in phospho-p65 and total p65 levels (Fig. 6G, H), corroborating GL’s action through this pathway.

Finally, we evaluated whether the GL 70% EtOH extract protects neurons against microglial neuroinflammatory responses using a transwell co-culture system. BV2 microglial cells (upper compartment) were pretreated with GL for 1 h, followed by LPS stimulation for 24 h. The conditioned medium was then applied to PC12 neuronal cells (lower compartment). An MTT assay revealed that GL treatment dose-dependently increased the viability of PC12 cells exposed to this microglia-conditioned medium (Fig. 5E, F).

Together, these results demonstrate that GL possesses antioxidant and anti-inflammatory properties and inhibits microglial activation by suppressing the NF-κB/STAT3 signaling pathway.

## Discussion

To our knowledge, this is the first study to demonstrate that *Ganoderma lucidum* (GL) can serve as a preventive and early-intervention agent against EAE/MS pathogenesis. Using LC–MS/MS, we identified 19 bioactive compounds in the GL extract, primarily ganoderic acids, providing a detailed chemical profile of the material. Through early pretreatment and preventive protocols, GL administration (0.32 mg/g/day) significantly delayed disease peak onset, alleviated clinical symptoms, and reduced both cumulative and maximum clinical scores in active EAE mice. Furthermore, GL markedly decreased inflammatory infiltration and demyelination in spinal cord lesions. Mechanistically, GL inhibited microglial activation and promoted a shift in polarization, exerting its effects via suppression of the NF-κB/STAT3 signaling pathway. This study offers novel insights into the active components and therapeutic mechanisms of *Ganoderma lucidum* in neuroinflammatory disease.

Multiple sclerosis (MS) involves demyelination, inflammation, axonal degeneration, and gliosis, with focal inflammatory lesions in the relapsing phase and diffuse inflammation with neurodegeneration in progression [31]. While FDA-approved therapies can alleviate symptoms, they are limited by side effects and resistance [9, 29], and no prophylactic health supplement currently exists to prevent MS relapse or progression. Natural products like polyphenols and saponins are studied for anti-inflammatory effects but typically act through single pathways (e.g., Nrf2, TLR4) and target existing disease rather than long-term prevention [26, 43]. In contrast, GL contains polysaccharides and terpenoids that may act synergistically to target multiple pathways for anti-inflammation, neuroprotection, and antioxidation from early, pre-symptomatic stages. Supported by our acute toxicity results and long-term safety data in literature [41], GL demonstrates promise as a safe, long-term supplement. Furthermore, GL pretreatment significantly reduced spinal cord infiltration of CD3⁺ T cells, CD11b⁺ macrophages/microglia, and CD45⁺ leukocytes, and attenuated demyelination in EAE mice, confirming its anti-neuroinflammatory and neuroprotective potential.

Microglia play a dual role in neurodegenerative pathology, capable of polarizing into pro-inflammatory M1 or anti-inflammatory M2 phenotypes in response to environmental cues. While M1 activation aggravates neuroinflammation and neuronal injury through the release of cytokines and free radicals, M2 polarization supports anti-inflammatory and neuroprotective functions [15]. Phagocytosis is essential for tissue homeostasis,however, dysregulated phagocytic activity can exacerbate demyelination and oxidative stress [13]. In MS/EAE, impaired microglial polarization and excessive phagocytosis contribute to neuroinflammation and neurodegeneration [2, 5]. Shifting the balance from M1 to M2 phenotypes can restore homeostasis and improve outcomes [8]. In this study, GL attenuated excessive microglial phagocytosis, upregulated the M2 marker CD206, and downregulated the M1 marker CD86 in both in vivo and in vitro models. By promoting an M2-like phenotype, GL likely contributes to the resolution of neuroinflammation and the preservation of neuronal function in EAE/MS.

Activated microglia are central to neuroinflammation and neurodegeneration in EAE/MS, in part by inducing oxidative stress and releasing inflammatory factors [18, 36]. In vitro, GL demonstrated antioxidant and anti-inflammatory effects in LPS-stimulated BV2 microglial cells. It significantly reduced levels of reactive oxygen and nitrogen species (O₂·⁻, NO·, and ONOO⁻) and downregulated the expression of pro-inflammatory proteins, including IL-1β, TNF-α, IL-6, COX2, and iNOS. In our previous studies, ONOO⁻ is proved to lead mitophagy activation and the disruption of the Treg/Th17 balance, exacerbating neuroinflammation in EAE [24, 40]. To assess whether GL could protect neurons from microglia-mediated damage, we employed a Transwell co-culture system with BV2 and PC12 cells. While LPS-activated BV2 cells induced PC12 death, GL pretreatment protected PC12 neurons from this oxidative and inflammatory injury. These findings indicate that GL mitigates microglial-associated oxidative and nitrative stress, thereby potentially preventing inflammatory-mediated neuronal damage in EAE/MS.

NF-κB/STAT3 signaling pathway plays essential roles in inflammation and oxidative damage [27]. Activation of the NF-κB/STAT3 pathway triggers microglia to release pro-inflammatory cytokines and ROS/RNS, leading to neuronal death and neurodegeneration [14]. Our data revealed that GL significantly reduced the phosphorylation and nuclear translocation of p65 and STAT3 phosphorylation in the microglial cells. These findings highlight that the NF-κB/STAT3 pathway might be one of the underlying mechanisms contributing to the GL’s neuroprotection.

For active compounds in GL extract, we mainly identified ganoderic acids which are the specific compounds existed in GL. Of the 19 ganoderic acids identified, ganoderic acid A has been shown to suppress pro-inflammatory cytokines and mitigate LPS-induced microglial activation and neurotoxicity [19]. It also inhibits the Rho/ROCK/NF-κB pathway, alleviating conditions such as acute lung injury [39]. We will further identify the anti-inflammatory and antioxidant properties of those ganoderic acids separately. Notably, GL also contains other natural compounds like polyphenols and saponins, etc. Those commonly existed active compounds in medicinal plants might also contribute to the anti-inflammatory and antioxidant properties for neuroprotection. These polyphenols and saponins might play independently and/or synergistically roles in attenuating EAE/MS pathology, highlighting the unique pharmacological features of GL. The chemical profiles of GL extract warrant further investigation, and the potential synergistic effects of its multiple active compounds remain to be elucidated.

In conclusion, *Ganoderma lucidum* is a promising medicinal plant for inhibiting inflammatory infiltration and demyelination and alleviating the severity of neural deficit symptoms and pathogenesis. The antioxidant and anti-inflammatory properties contribute to the underlying mechanisms of inhibiting microglial activation and phagocytosis and modulating NF-κB/STAT3 signaling pathways.

## Supplementary Information

Supplementary material 1. Supplementary Figure 1. Fingerprint analysis of GL by LC-MS/MS. Representative total negative iron chromatograms of GL extractions in LC-MS/MS analysis. Supplementary Figure 2. GL treatment mitigates active EAE symptoms by using the prevention protocol. Mice were orally administered with vehicleor GLstarting from 2 days post-EAE induction. Accumulative clinical scores up to 30 dpi were calculated. Data are presented as Mean ± S.E.M., *p < 0.05, **p < 0.01, or ****p < 0.0001 versus EAE group. Statistical analysis was performed using one-way ANOVA. Supplementary Figure 3. GL reduces inflammation in the spinal cord of EAE mice at 11, 18, and 30 dpi with the prevention protocol. **A** Representative H&E staining of spinal cord sectionsfrom EAE mice at 11, 18, and 30 dpi. **B** Immunohistochemical staining of CD45 for leukocyte infiltration into the CNS. Lumbar spinal cords were collected at 30 dpi.. **C** Statistical analysis reveals that GL pretreatmentattenuates CD45 positive staining compared with the EAE vehicle group. Data were presented as Mean ± S.E.M., **p < 0.01, versus the EAE control group. Statistical analysis was performed using the Student's t-test. Supplementary Figure 4. GL treatment with the prevention protocol inhibits inflammatory infiltration including CD3 positive T cells, CD11b positive macrophages/microglia, and CD45 positive leucocytes. Immunofluorescence staining of lumbar spinal cordsfrom EAE mice. **A **Representative immunohistochemical staining of CD3, CD11b, CD45 and DAPI. **B** Statistical analysis reveals that GL treatment inhibited the CD3, CD11b, and CD45 positive staining cells. Data are presented as Mean ± S.E.M., ## p < 0.05, ### p<0.01, versus sham control group; *p < 0.05, **p < 0.01, or ****p < 0.0001 versus EAE group; Statistical analysis was determined using one-way ANOVA. Supplementary Figure 5. Safety assessment of oral administration of GL at a dosage of 0.96 mg/g body weight in normal and EAE mice via histopathological and serum biochemical analyses. **A–C** Histopathological and serum biochemical evaluations at 11 days after GL treatment. **A** Hematoxylin-eosinstaining of kidney tissues, **B** H&E staining of liver tissues, andserum levels of AST, ALT, BUN, CRE, and UA in the control group, GL-treated normal group, EAE group and EAE + GL-treated group. No significant histopathological lesions or functional abnormalities were observed in the liver and kidney of mice in each group. **D–F** Histopathological and serum biochemical evaluations at 30 dpi. **D** H&E staining of kidney tissues, **E** H&E staining of liver tissues, andserum levels of AST, ALT, BUN, CRE and UA in the corresponding groups. Oral GL administration did not induce overt histopathological toxicity or functional impairment in the liver and kidney of normal or EAE mice at 30dpi. Supplementary Figure 6. GL suppresses nuclear translocation of p65 as detected by immunofluorescence staining. BV2 cells were pre-incubated with GLor sham for 1 h prior to LPS stimulationfor 24 h. Immunofluorescence staining was performed to visualize the subcellular localization of p65 in BV2 cells from control and GL-treated groups. Nuclei were counterstained with DAPI, and the fluorescence intensity of nuclear p65 was quantified to assess the translocation efficiency. Supplementary Figure 7. Full-length original Western blot images of target proteins and corresponding molecular weight markers Note: All Western blot experiments utilized three or more independent biological replicates. While standard imaging conditions typically limited the visualization of molecular weight markers, some nonspecific signals were noted in partial blots. Each target protein band discussed in the main text underwent thorough verification to rule out nonspecific interference and confirm data accuracy.

Supplementary material 2

## Data Availability

No datasets were generated or analysed during the current study.

## Abbreviations

BG-12: Dimethyl fumarate
CNS: Central nervous system
COX2: Cyclooxygenase 2
DAF-2 DA: 4,5-Diaminofluorescein diacetate
dpi: Day post-immunization
EAE: Experimental autoimmune encephalomyelitis
Het: Hydroethidine
IF: Immunofluorescence
IHC: Immunohistochemistry
IL-1β: Interleukin-1β
IL–6: Interleukin–6
LFB: Luxol fast blue
MyD88: Myeloid differentiation primary response 88
MOG_35-55_: Myelin oligodendrocyte glycoprotein_35-55_
MS: Multiple Sclerosis
MTT: 3-(4,5-Dimethylthia-zol-2-yl)-2,5-diphenyltetrazolium bromide
NF-κB: Nuclear factor-κB
NO: Nitric oxide
O_2_^−^: Superoxide anion
ONOO^−^: Peroxynitrite
p-IκBα: Phosphorylated inhibitor of κBα
RNS: Reactive nitrogen species
ROS: Reactive oxygen species
STAT3: Signal transducer and activator of transcription 3
TNF–α: Tumor necrosis factor-α
GL: *Ganoderma lucidum*
GLP: *Ganoderma lucidum* polysaccharides
