# Supplementary material for: Ganoderma lucidum mediates microglial polarization and ameliorates experimental autoimmune encephalomyelitis by reducing oxidative stress and inhibiting NF-κB/STAT3 pathway
Source: Chin Med. 2026 Apr 10;21:114. doi: 10.1186/s13020-026-01327-x (PMC13067805; doi:10.1186/s13020-026-01327-x)

Supplementary Figure 1 (Fig. S1)

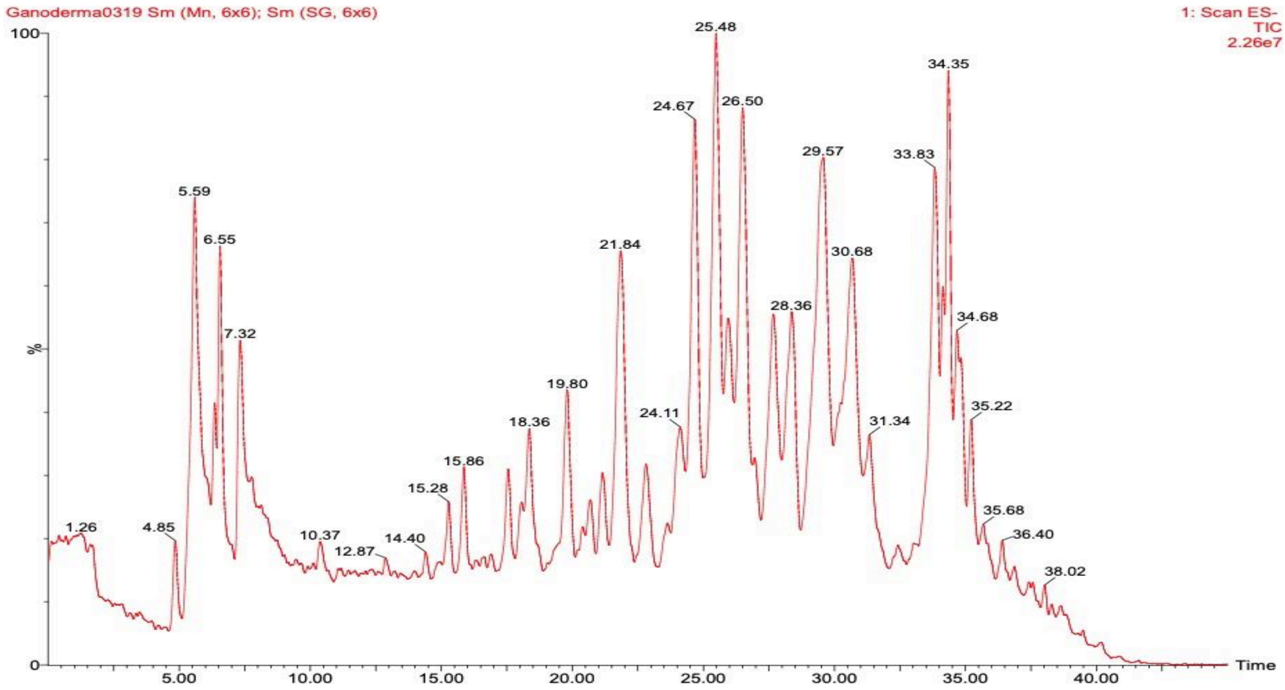

Supplementary Figure2 (Fig. S2)

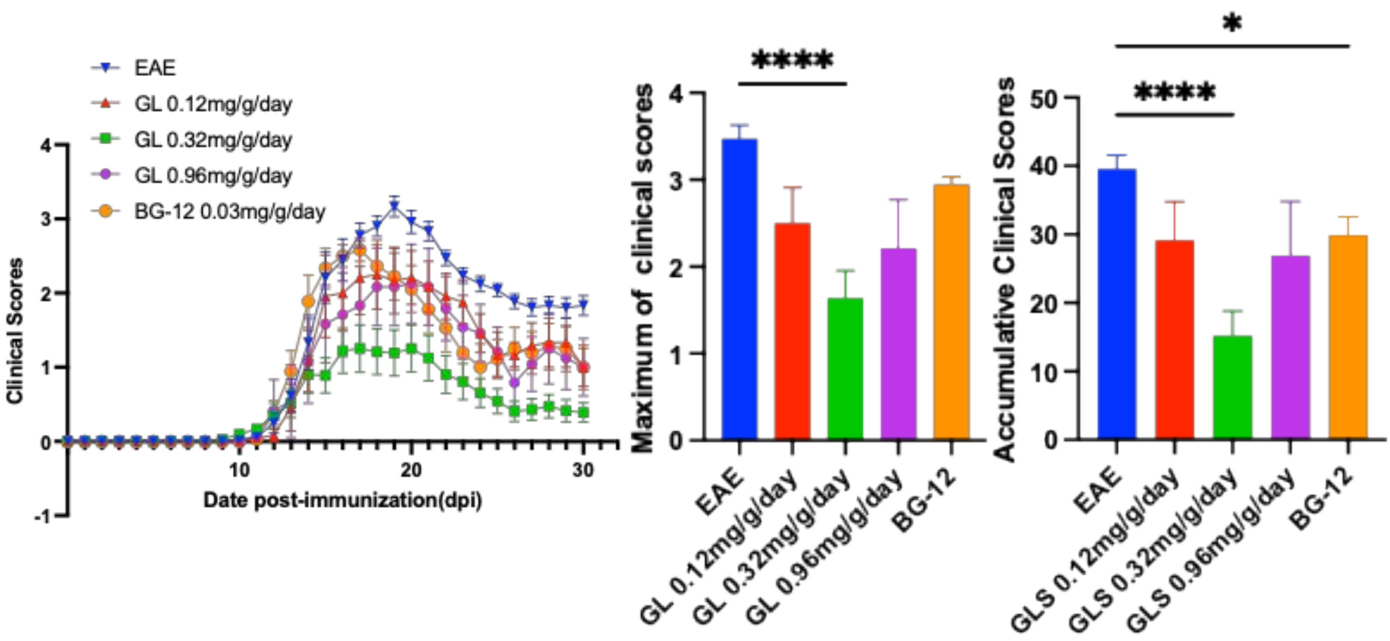

Supplementary Figure 3 (Fig. S3)

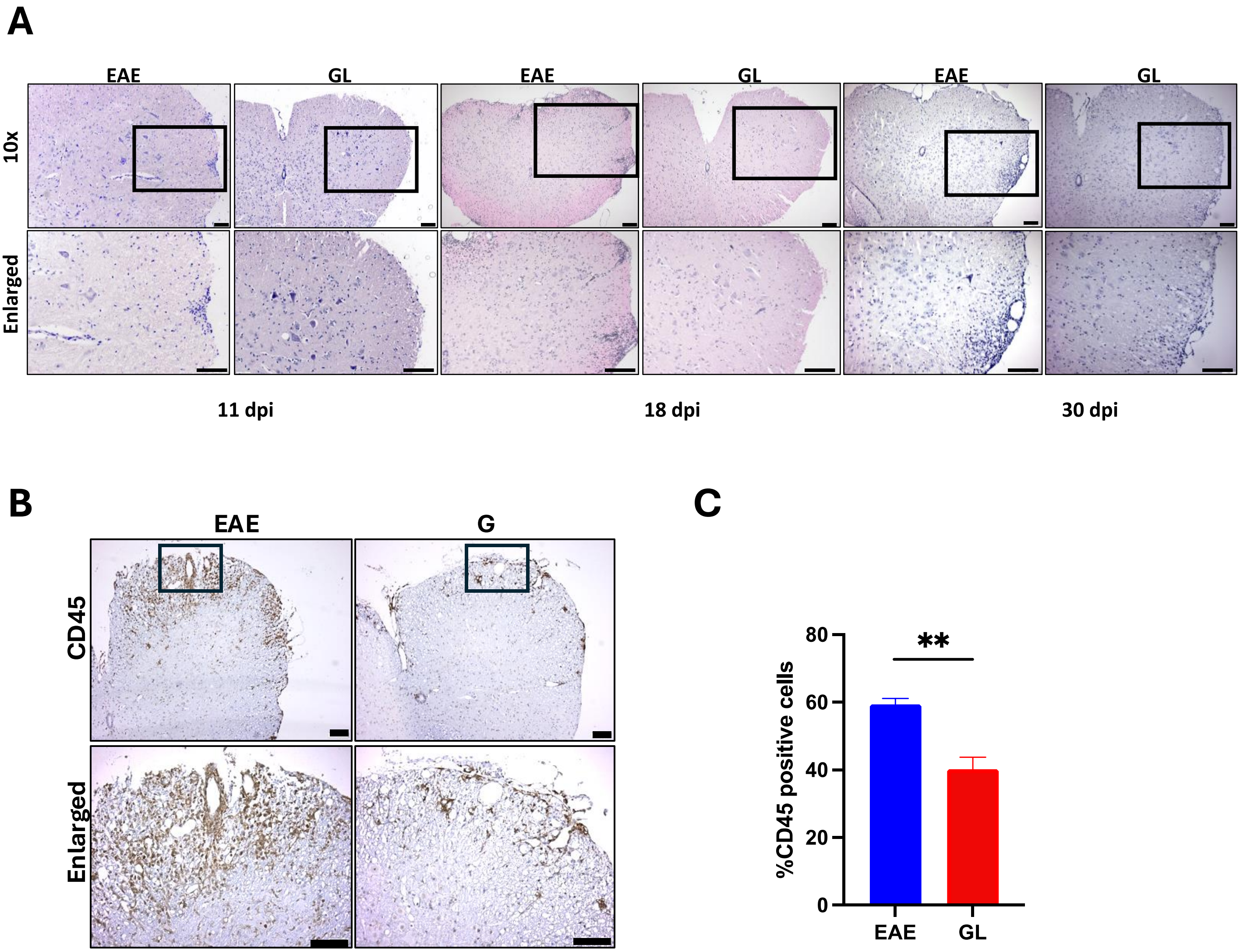

Supplementary Figure 4 (Fig. S4)

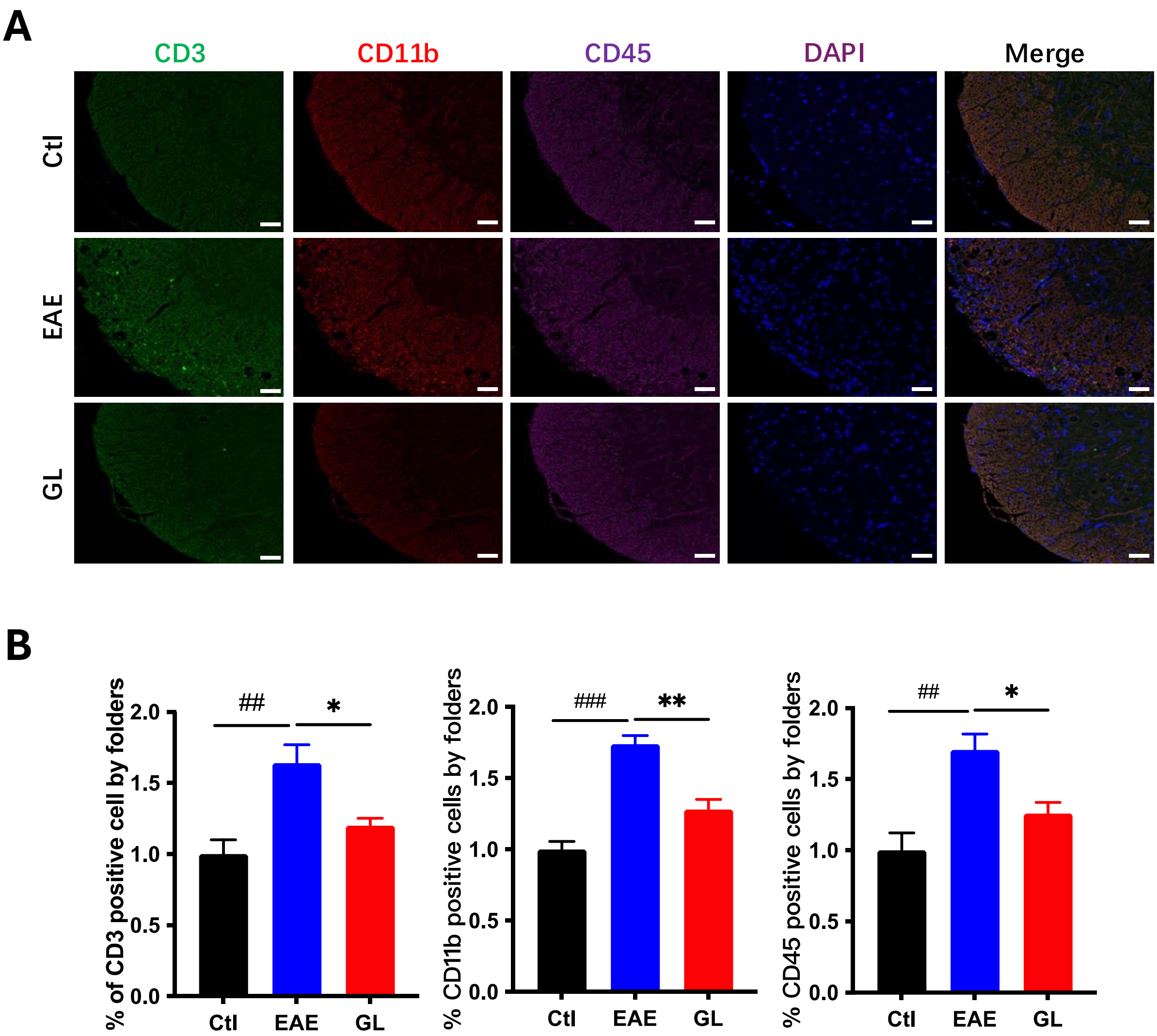

Supplementary Figure 5 (Fig. S5)

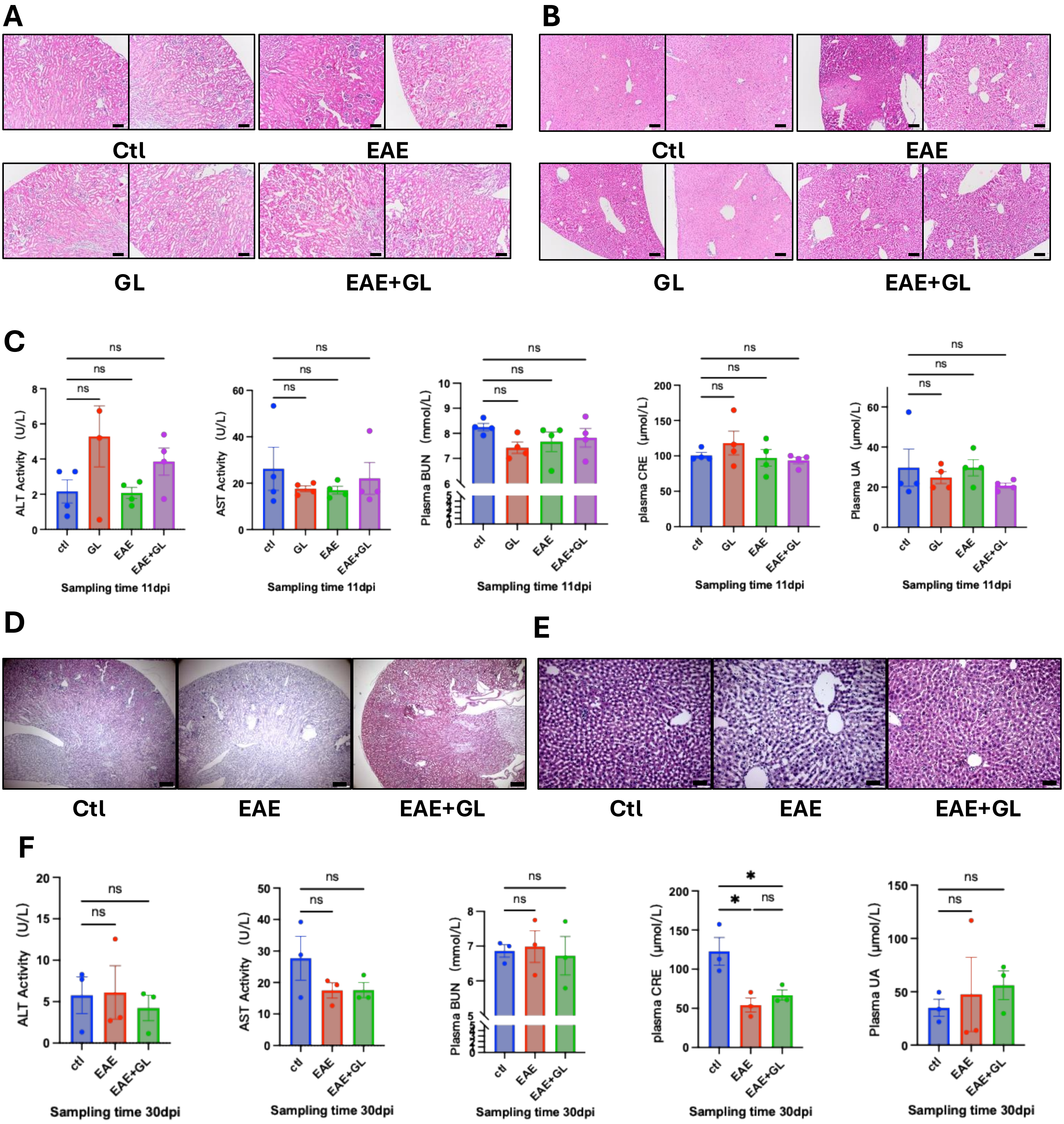

Supplementary Figure 6 (Fig. S6)

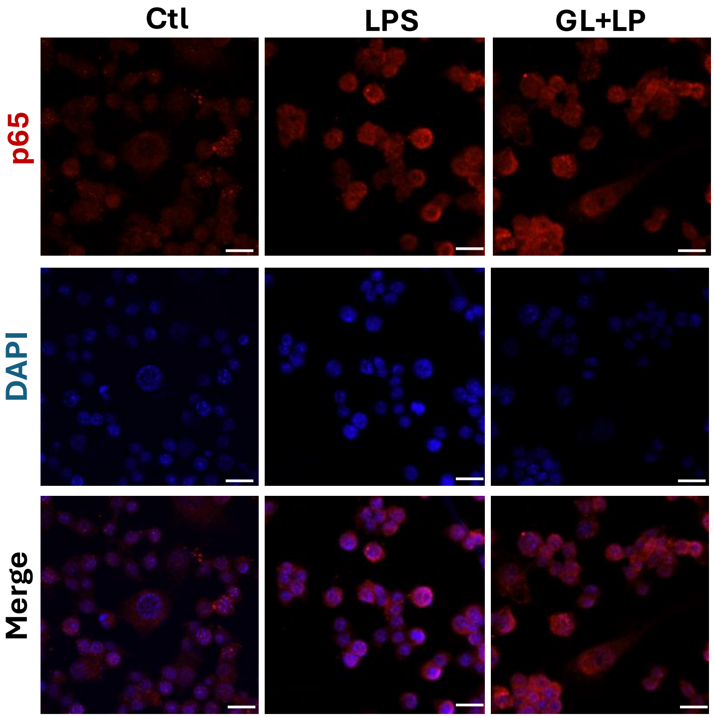

Supplementary Figure 7 (Fig. S7A)

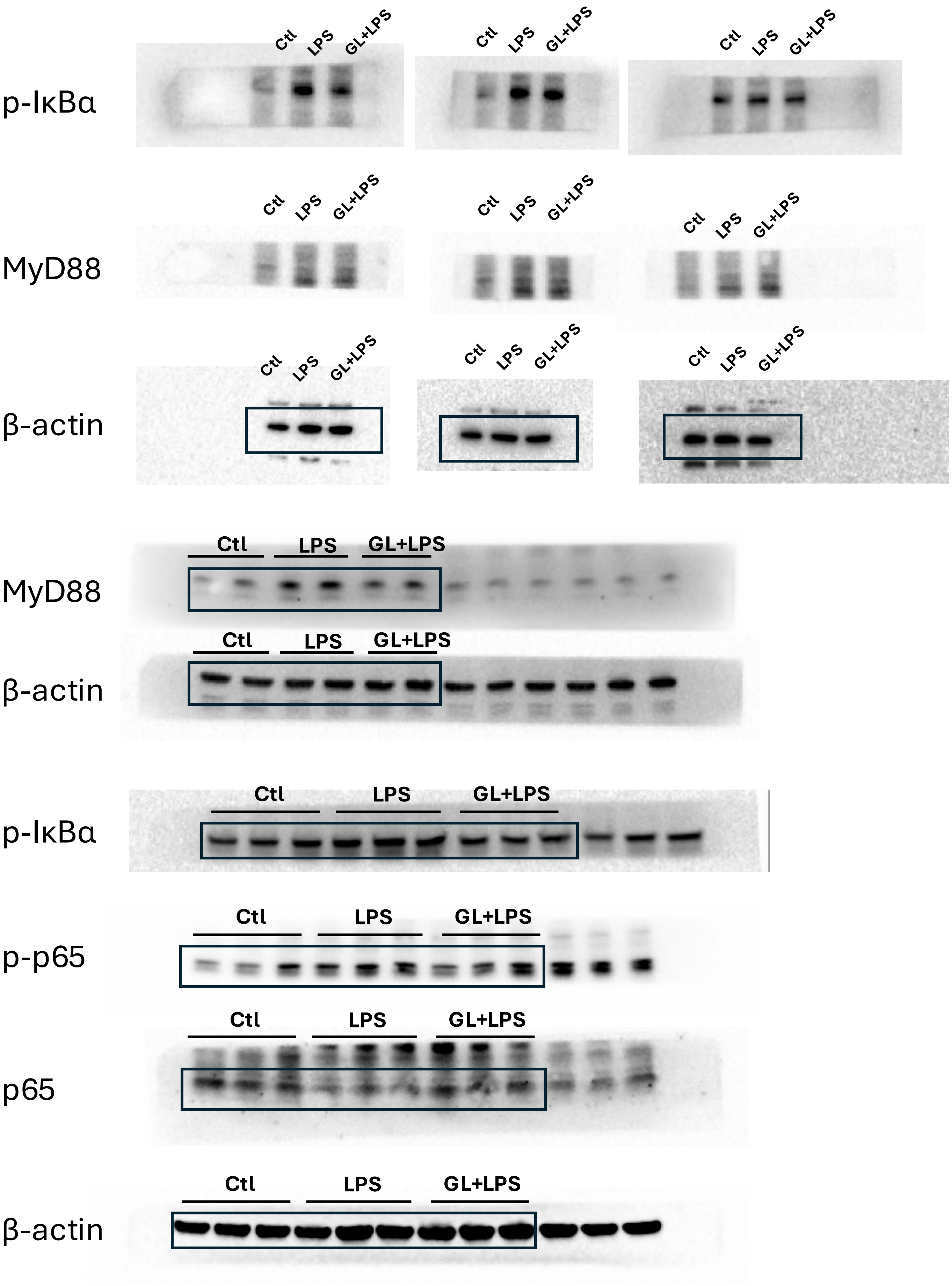

Supplementary Figure 6 (Fig. SB)

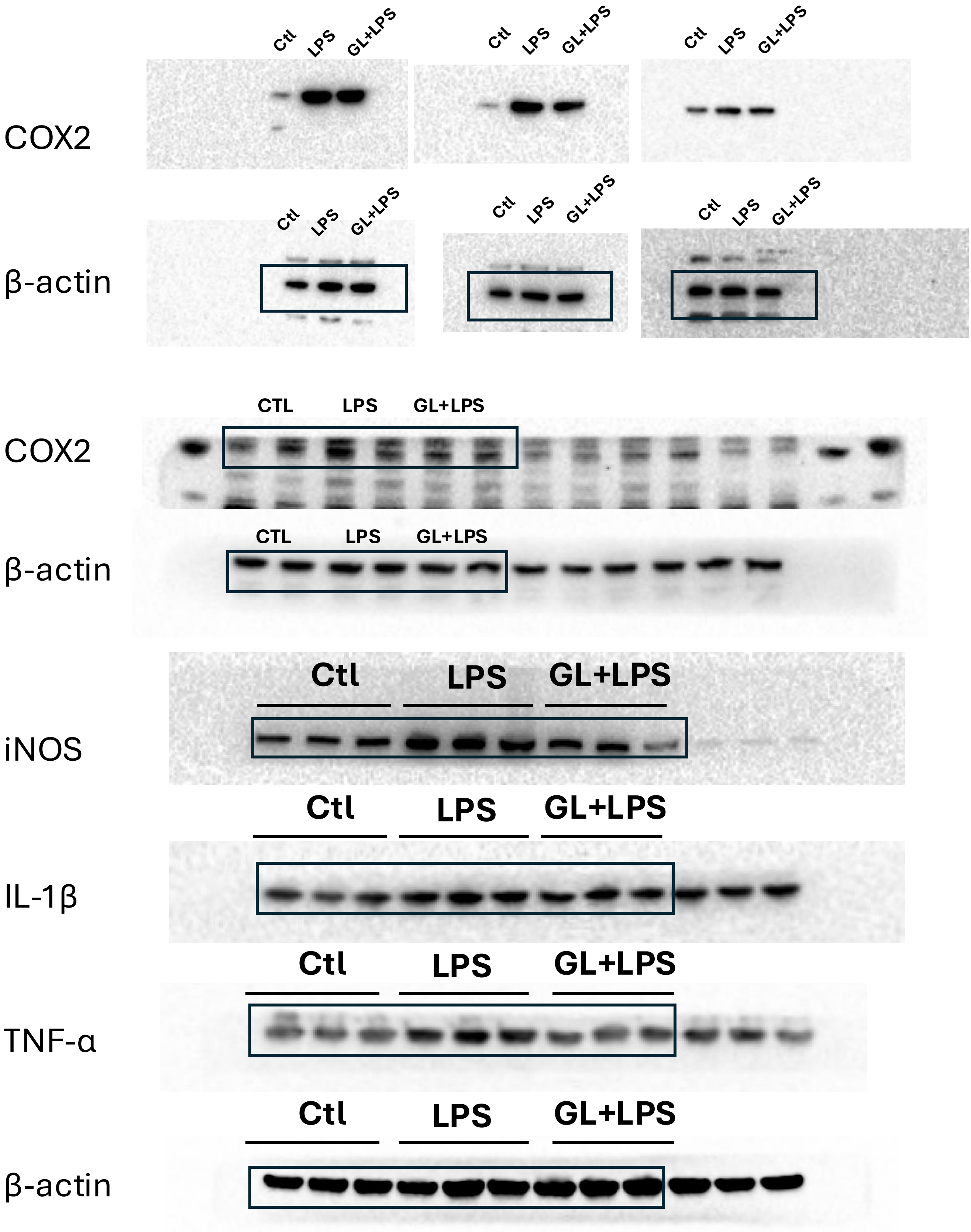

Supplementary Figure 6 (Fig. S7C)

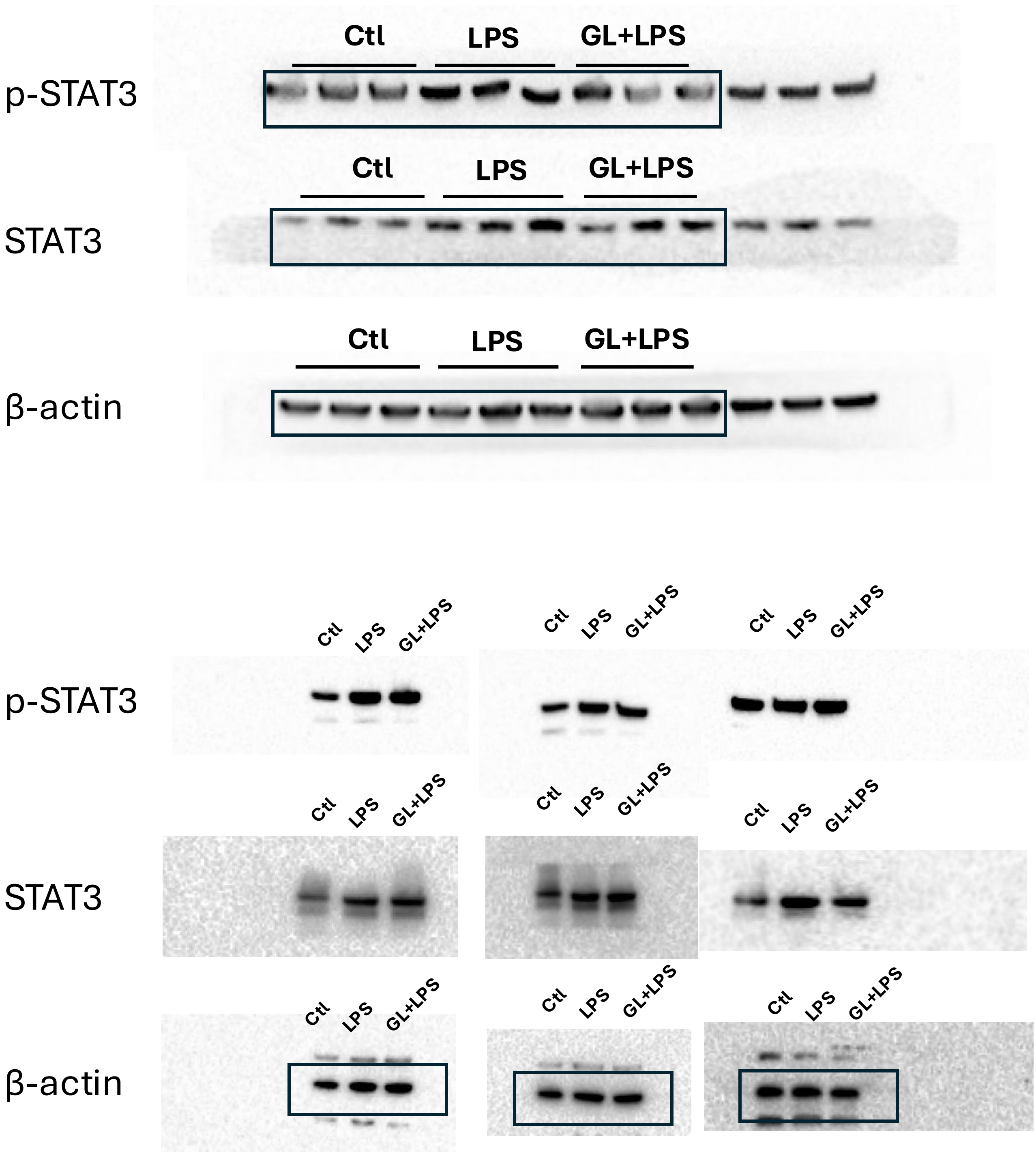

Supplementary Figure 6 (Fig. S7D)

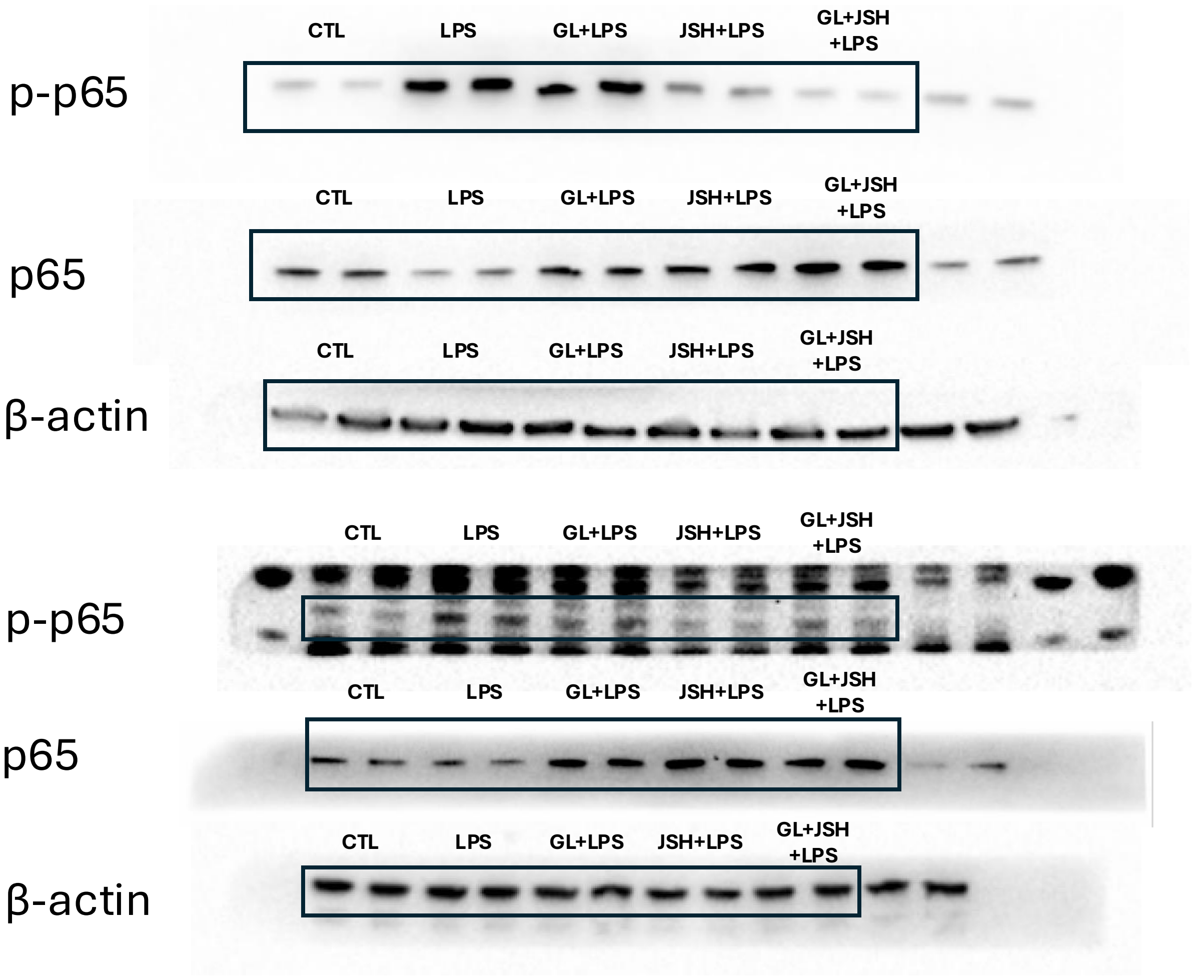

Supplement: Supplementary file 1 — Supplementary material 1. Supplementary Figure 1. Fingerprint analysis of GL by LC-MS/MS. Representative total negative iron chromatograms of GL extractions in LC-MS/MS analysis. Supplementary Figure 2. GL treatment mitigates active EAE symptoms by using the prevention protocol. Mice were orally administered with vehicleor GLstarting from 2 days post-EAE induction. Accumulative clinical scores up to 30 dpi were calculated. Data are presented as Mean ± S.E.M., *p < 0.05, **p < 0.01, or ****p < 0.0001 versus EAE group. Statistical analysis was performed using one-way ANOVA. Supplementary Figure 3. GL reduces inflammation in the spinal cord of EAE mice at 11, 18, and 30 dpi with the prevention protocol. A Representative H&E staining of spinal cord sectionsfrom EAE mice at 11, 18, and 30 dpi. B Immunohistochemical staining of CD45 for leukocyte infiltration into the CNS. Lumbar spinal cords were collected at 30 dpi.. C Statistical analysis reveals that GL pretreatmentattenuates CD45 positive staining compared with the EAE vehicle group. Data were presented as Mean ± S.E.M., **p < 0.01, versus the EAE control group. Statistical analysis was performed using the Student's t-test. Supplementary Figure 4. GL treatment with the prevention protocol inhibits inflammatory infiltration including CD3 positive T cells, CD11b positive macrophages/microglia, and CD45 positive leucocytes. Immunofluorescence staining of lumbar spinal cordsfrom EAE mice. A Representative immunohistochemical staining of CD3, CD11b, CD45 and DAPI. B Statistical analysis reveals that GL treatment inhibited the CD3, CD11b, and CD45 positive staining cells. Data are presented as Mean ± S.E.M., ## p < 0.05, ### p<0.01, versus sham control group; *p < 0.05, **p < 0.01, or ****p < 0.0001 versus EAE group; Statistical analysis was determined using one-way ANOVA. Supplementary Figure 5. Safety assessment of oral administration of GL at a dosage of 0.96 mg/g body weight in normal and EAE mice via histopathological [file 13020_2026_1327_MOESM1_ESM.pdf]
