# Supplementary material for: Ganoderma lucidum mediates microglial polarization and ameliorates experimental autoimmune encephalomyelitis by reducing oxidative stress and inhibiting NF-κB/STAT3 pathway
Source: Chin Med. 2026 Apr 10;21:114. doi: 10.1186/s13020-026-01327-x (PMC13067805; doi:10.1186/s13020-026-01327-x)
Supplement: Supplementary file 2 — Supplementary material 2 [file 13020_2026_1327_MOESM2_ESM.docx]

**Supplementary Table 1. Compounds identified in GL extract**

| **No.** | **RT (min)** | **Declustering potential (V)** | **Collision Energy (V)** | **Formula** | **Identity** |
| --- | --- | --- | --- | --- | --- |
| 1 | 7.34 | -80 | -15 | C_32_H_42_O_9_ | 3-O-Acetyl ganoderic acid H |
| 2 | 12.87 | -80 | -15 | C_30_H_42_O_7_ | Ganoderenic acid G |
| 3 | 18.36 | -80 | -15 | C_30_H_40_O_7_ | Ganoderic acid N |
| 4 | 19.8 | -80 | -15 | C_27_H_40_O_6_ | Ganoderic acid G |
| 5 | 19.96 | -80 | -15 | C_30_H_44_O_8_ | Ganoderic acid I |
| 6 | 20.6 | -80 | -15 | C_30_H_44_O_7_ | Ganoderic acid C6 |
| 7 | 21.13 | -80 | -15 | C_30_H_42_O_8_ | Ganoderenic acid C |
| 8 | 21.84 | -80 | -15 | C_27_H_36_O_6_ | Ganoderic acid C2 |
| 9 | 24.19 | -80 | -15 | C_30_H_44_O_8_ | Lucidenic acid N |
| 10 | 24.67 | -80 | -15 | C_30_H_40_O_7_ | Ganoderenic acid H |
| 11 | 25.48 | -80 | -15 | C_30_H_42_O_8_ | Ganoderic acid B |
| 12 | 29.57 | -80 | -15 | C_32_H_44_O_9_ | Ganoderic acid A |
| 13 | 30.68 | -80 | -15 | C_30_H_44_O_7_ | Ganoderic acid H |
| 14 | 31.34 | -80 | -15 | C_30_H_44_O_7_ | Ganoderenic acid B |
| 15 | 33.83 | -80 | -15 | C_30_H_38_O_7_/C_30_H_46_O_7_ | Ganoderic acid E/ Ganoderenic acid D |
| 16 | 34.35 | -80 | -15 | C_30_H_40_O_7_ | Ganoderic acid D |
| 17 | 34.68 | -80 | -15 | C_30_H38 O7/C_30_H_46_O_7_ | Ganoderic acid E/ Ganoderenic acid D |
| 18 | 35.22 | -80 | -15 | C_30_H_42_O7/C_34_H_46_O_10_ | Ganoderenic acid F/ Ganoderic acid F |
| 19 | 35.22 | -80 | -15 | C_30_H_42_O7/C_34_H_46_O_10_ | Ganoderenic acid F/ Ganoderic acid F |
